# Occupational differences in sickness presenteeism in the baseline survey of the German National Cohort (NAKO)

**DOI:** 10.1186/s12995-026-00529-1

**Published:** 2026-09-25

**Authors:** Thi-Ngoc-Tu Nguyen, Melanie Schubert, René Mauer, Marvin Reuter, Nico Dragano, Andreas Seidler

**Affiliations:** 1 Department of Occupational and Social Medicine, Public Health, Institute and Policlinic of Occupational and Social Medicine, Faculty of Medicine Carl Gustav Carus, Dresden University of Technology, 01307 Dresden, Germany; 2 Institute for Medical Informatics and Biometry, Faculty of Medicine Carl Gustav Carus, Dresden University of Technology, 01307 Dresden, Germany; 3 Junior Professorship for Sociology, esp. Work and Health, Institute for Sociology, Faculty of Social Sciences, Economics and Business Administration, 96045 Bamberg, Germany; 4 Institute of Medical Sociology, Centre for Health and Society, Medical Faculty, University of Duesseldorf, 40225 Duesseldorf, Germany

**Keywords:** Sickness presenteeism, Occupations, Employees, NAKO, Germany

## Abstract

**Background:**

This study examines the relationship between occupation and sickness presenteeism (SP) among German employees. In addition, it investigates occupation-specific differences in SP according to supervisory or managerial responsibilities.

**Methods:**

A total of 86,855 employees (average age: 45.7 ± 10.5, 48.3% men, and 51.7% women) from the German National Cohort (NAKO) who had reported illness at least once in the past 12 months were included in this cross-sectional study (response: 15.6%). Data were collected between 2014 and 2019. Occupations were grouped into 14 segments based on the German Classification of Occupations 2010. SP was defined as attending work despite illness at least once within the past 12 months. Robust Poisson regression analyses were conducted to estimate prevalence ratios (PRs) and 95% confidence intervals (CIs) for SP, comparing each occupational segment with all other occupations combined as the reference group. Additional analyses compared SP between employees with and without supervisory or managerial responsibilities overall and within each occupational segment, and by sex.

**Results:**

Among employees who had been ill at least once in the past 12 months, 76.4% reported SP. The highest prevalence of SP was observed in healthcare, social and cultural service occupations, trade, and food industry and hospitality. Overall, employees with supervisory or managerial responsibilities had a higher SP prevalence than those without such responsibilities (PR = 1.08; 95% CI = 1.07–1.10). This association was stronger among men (PR = 1.11; 95% CI = 1.09–1.12) than among women (PR = 1.04; 95% CI = 1.02–1.06). In general, the elevated prevalence of SP among supervisors and managers was not confined to specific occupational segments. Rather, certain occupational segments exhibited consistently higher SP prevalence regardless of hierarchical position.

**Conclusions:**

Among German employees, SP prevalence varies notably across occupations, with above-average levels in healthcare, social and cultural service, trade, and food industry and hospitality. Supervisory and managerial responsibilities were associated with a modestly higher prevalence of SP, with a stronger association among men than women. However, occupation-specific patterns were broadly similar across hierarchical groups, indicating that SP is likely influenced by a combination of occupational context and hierarchical position.

**Supplementary Information:**

The online version contains supplementary material available at https://doi.org/10.1186/s12995-026-00529-1.

## Background

Absenteeism, which refers to non-attendance at work due to illness, has consistently been a concern due to its detrimental economic impact [1]. The ‘opposite’, sickness presenteeism (SP), which is defined as the phenomenon of attending work despite health problems, also receives increasing attention due to its high prevalence and negative effects on health, the healthcare system, and the economy [2–5]. A review of 19 studies from 15 countries found that between 30% and over 90% of workers reported at least one episode of SP within a 6– to 12–month period [4]. In Germany, around 57% of employees reported going to work despite having health complaints at least once in the last 12 months [6]. A 2010 survey in Europe found that 45% of men and 41% of women had worked while sick at least one day in the previous 12 months [7]. Kinman [8], suggested that the prevalence of SP might continue to rise; however, more recent research indicates a decline in SP following the COVID-19 pandemic [9].

SP can have a negative impact on individual physical and mental health [5]. The findings of a prospective cohort study suggest that reporting SP at least five times a year is associated with a 2.53-fold higher risk of poor health and a 2.67-fold higher risk of physical complaints in the following year among Swedish employees [10]. In addition, a two-year longitudinal study in Denmark found that people who worked despite sickness for more than eight days at baseline had a 2.93 times higher risk of depression at follow-up than those who were on sick leave [2]. The long-term health effects of SP have also been demonstrated in a Swedish longitudinal study, which found that employees reporting sickness presenteeism had a higher risk of reporting fair or poor self-rated health at both the 18-month and 3-year follow-ups than employees who took sickness leave [11].

Many risk factors for SP have been investigated [12], including amongst others demographic and work-related characteristics. Studies suggest that women [13], middle-aged employees [13–15], and those with higher levels of education [16] are at a higher risk of SP. A higher risk of SP was also found among employees with perceived job insecurity [17–19], as well as among employees with long working hours [13, 14, 17], and those with insufficient or no support from their managers and colleagues [20].

The risk of SP might also differ between occupations. A recent review suggests that occupations in education and healthcare are at a higher risk of SP [21]. However, most studies have focused their research on specific occupations, such as nurses [22–24], police officers [25, 26], teachers [20, 27], or surgeons [28, 29]. Little research has been conducted including all occupations in a population-based study. The pioneering work of Aronsson and colleagues [30] used a population-based study sample from the labor market survey in Sweden. They observed the highest SP prevalences for nursing and midwifery professions, and care workers in nursing homes, as well as for compulsory school teachers and pre-school/primary educationalists. To date, population-based studies examining the association between occupation and SP in large and diverse working populations remain scarce. The present study contributes to this limited body of evidence by providing population-based findings for the German working population.

Additionally, one group that may be particularly prone to SP are managers [17, 21]. Managerial roles are typically characterized by high levels of responsibility, decision-making authority, and accountability for team and organizational outcomes. These features often result in low substitutability, meaning that managers may perceive their absence as especially disruptive. Early theoretical work suggests that employees in positions with high indispensability are less likely to be absent [31]. In line with this, empirical research has identified job demands, time pressure, and lack of replacement options as key drivers of working while ill [22, 30, 32, 33].

However, the extent to which managers engage in SP may not be uniform across occupations, as work contexts differ substantially in terms of task interdependence, professional norms, job autonomy, and organizational constraints. In healthcare settings, for example, managers often face strong pressure to remain present despite illness because their absence can disrupt workflows and burden their teams [8]. In contrast, managers in occupations characterized by higher job autonomy and more flexible work organization may face a lower risk of SP, as tasks can be adjusted or redistributed more easily [32]. Despite these important contextual differences, research explicitly examining managerial SP across different occupations remains limited.

Thus, this study aims to investigate the relationship between different occupational segments and the prevalence of SP among employees. Within this aim, this study also examines the effect of managerial/supervisory positions on SP prevalence across occupational segments. The research questions are: What is the relationship between occupational segments and SP among employees, and which professions are most likely to be affected by SP? Is there an increased SP prevalence among employees in supervisory and management positions across all occupational segments, or are there differences specific to certain segments?

## Methods

The aim of this study is to investigate the association between occupational segments and SP among German employees and to determine whether supervisory and managerial positions are associated with higher SP prevalence across occupational segments or whether these associations vary between segments. In Germany, employees who are unable to work due to illness are entitled to continued payment of their full salary by their employer for up to six weeks (*Entgeltfortzahlung*), provided they submit a medical certificate of incapacity for work (*Arbeitsunfähigkeitsbescheinigung*). If the same episode of illness persists beyond six weeks, statutory health insurance provides sickness benefit (*Krankengeld*), which generally amounts to approximately 70% of the employee’s gross salary (subject to statutory limits).

### Data and study population

This cross-sectional study uses the baseline dataset of the German National Cohort (NAKO), a large population-based and prospective cohort study in Germany. The baseline survey was conducted between 2014 and 2019 across 18 study centers in 16 study regions of Germany [34]. Participants were randomly selected from age- and sex-stratified samples from resident registers of the study areas. During recruitment, 1,364,918 individuals aged 20–69 years were randomly selected from local population registers. After excluding ineligible individuals (e.g., deceased persons, people who had moved away from the study region, or individuals who did not meet the eligibility criteria), 1,316,055 persons remained eligible for participation. Of these, 205,414 completed the baseline examination, corresponding to an overall response proportion of 15.6%, although response varied considerably between study centers (7.6%–30.7%) [35]. Response was higher among women than men, increased with age, and was highest in rural areas compared with urban regions. The NAKO cohort includes a higher proportion of participants with high educational attainment and a lower proportion of participants with low and medium educational attainment compared to the general German population [35].

At baseline, participants provided information on their socio-demographics, work-related experiences, medical history, and health habits through face-to-face interviews and touchscreen surveys. For the current analyses, only participants who were employed at the time of the baseline survey were included (*n* = 130,786). Trainees, students, and self-employed individuals (with a total of *n* = 20,183) were excluded from the study sample, as their working conditions differ from those of other employees. In addition, only employees who reported illness (impairing professional activities) and/or sickness presence in the past 12 months were included in the analysis. Employees who fulfilled both of the following criteria were excluded from the analytical sample (*n* = 13,554):

1. Responded “*No*,* never”* or ”*Does not apply”* to the question *“Did you go to work in the past twelve months when you should have stayed at home due to an illness?*” and.
2. Reported zero days to the question “*How many calendar days in total were you ill in the past 12 months in such a way that you were unable to pursue your usual professional activities?*”.

**Fig. 1:**
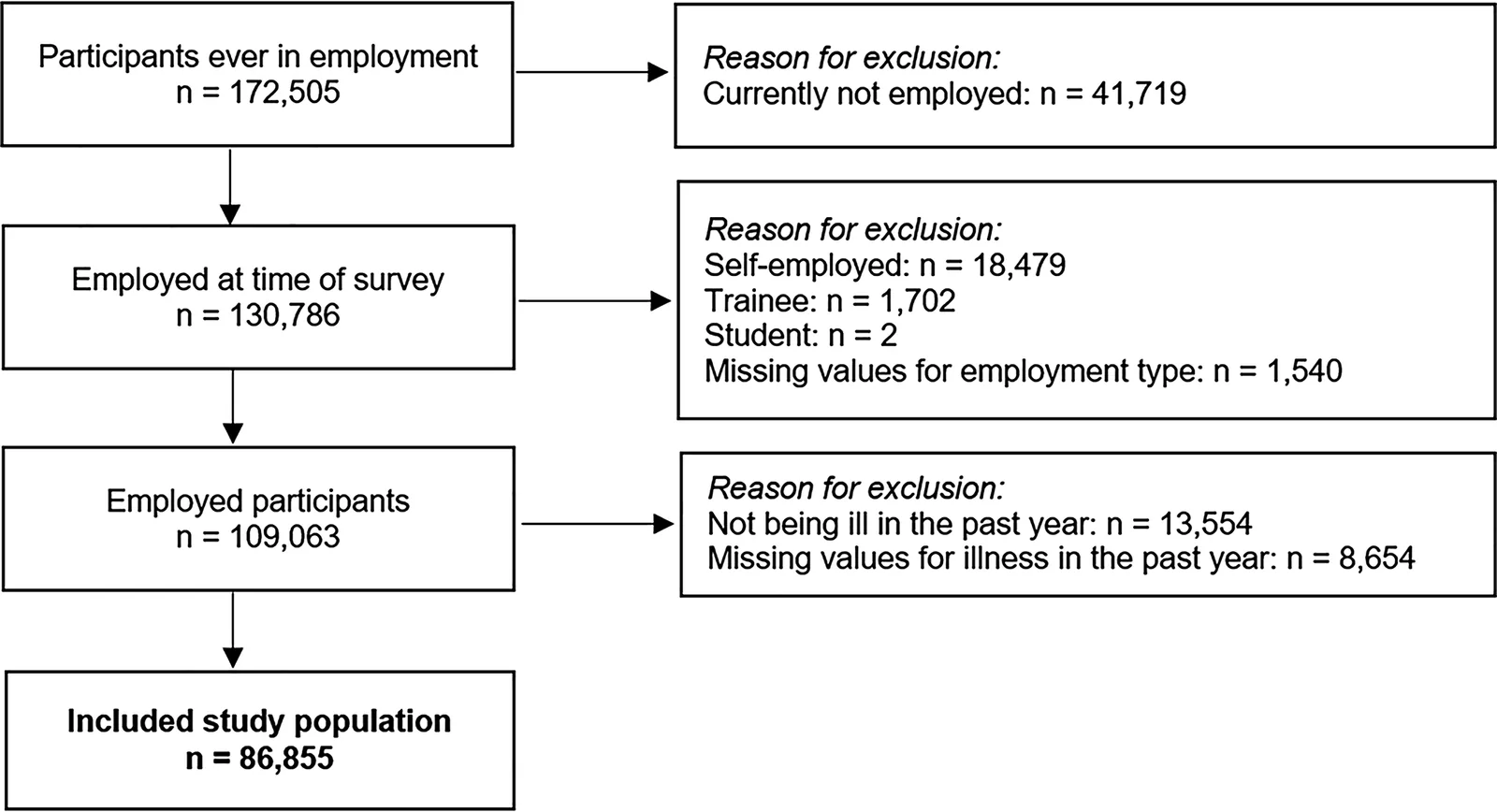
Flow chart

This approach was chosen because SP can only occur among employees who reported illness during the recall period and were therefore at risk of SP [13]. In the available NAKO dataset, the response options *‘No*,* never’* and *‘Does not apply’* could not be distinguished in the exported data and were therefore treated identically. To minimize potential misclassification, participants were excluded only if they additionally reported no days of illness impairing their work during the previous 12 months. The inclusion and exclusion criteria are summarized in Fig. 1. A total of 86,855 participants (48.3% men, 51.7% women) aged between 19 and 73 (with an average age of 45.7 ± 10.5 years) were included in the analysis.

### Variables

#### Occupational segments and occupational groups

Occupations were grouped based on the German Classification of Occupations (KldB) 2010) [36], a hierarchically structured five-digit code system. In this study, 14 occupational segments were distinguished, which were derived based on the occupational main group (first two digits of the KldB) and developed by the German Federal Employment Agency [37]. Each segment includes several occupational groups with related activities. A detailed overview of 14 occupational segments with their occupational groups is provided in Additional file 1. This procedure has been used before for the determination of occupation-specific risk, such as SARS-CoV-2 infection [38]. In addition to the analyses by occupational segment, supplementary analyses were conducted at the 3-digit occupational group level to provide a more detailed assessment of occupation-specific SP risks. The hierarchical position (non-supervisory/non-managerial employees versus supervisory/managerial employees) was defined based on the fourth digit of the KldB.

#### Sickness presenteeism (SP)

SP was assessed in NAKO using the following self-report question: ‘Did you go to work during the past twelve months when you should have stayed at home due to an illness?’. The possible responses were ‘Yes, once (1)’, ‘Yes, twice to five times (2)’, ‘Yes, more than five times (3)’, ‘No, never (4)’, and ‘Does not apply (5)’ [32]. Thus, SP was assessed as the frequency of occasions on which participants attended work despite illness rather than the total number of days worked while ill. Participants who answered ‘No, never (4)’ or ‘Does not apply (5)’ were considered ‘non-presentees’ (i.e., people who take sick leave when feeling unhealthy). Those who at least responded ‘once’ a year were identified as ‘presentees’ (people present at work despite illness). This definition aligns with the definition of previous research on SP [14, 19, 20, 23, 39–47].

#### Covariates

Three covariates were adjusted for as potential confounders of the relationship between occupational segments and SP: sex, age, and educational level, which affect both SP and occupational segments. Sex was categorized as men (1) and women (2). Age was analyzed as a continuous variable. In NAKO, the highest level of education was assigned according to the International Standard Classification of Education 1997 (ISCED 97) (levels 0–6) [48]. For this study, education was redefined according to the ISCED into the three levels: low (1) – included pre-primary, primary, and lower secondary level; moderate (2) – included upper secondary, post-secondary, and non-tertiary; and high (3) – included first stage of tertiary and second stage of tertiary education [49, 50].

Another control variable was health status, because employees who are more frequently ill may have greater chances to opt for SP, or those with more severe health conditions might be more restricted to sickness absence. Self-perceived health status was recorded in five categories: excellent (1), very good (2), good (3), less good (4), and poor (5).

Other sociodemographic variables that could be associated with occupational segments and SP are migration background, income, marital status, and the presence of children. Migration background was divided into no migration background (1) and migration background (2). Relative income was calculated based on the ratio between the equivalent net household income and the median equivalent net income in Germany in 2019, according to the European Union Statistics on Income and Living Conditions [51]. Net equivalent household income was determined based on the information on household size and net household income. Relative income was divided into five levels: Below 60%, indicating a risk of poverty, 60%–79%, 80%–99%, 100%–149%, and above 150%, representing high-income groups [52, 53]. In this study, the relative income was recoded into three categories: <60%, 60%–<150%, and ≥ 150%. Marital status was recorded in five categories: single (1), married living together (2), married living apart (3), divorced (4), and widowed (5). Information on children was collected in the NAKO study for the number of children under 14 living in the household. In this study, all persons who reported having at least one child were classified as ‘Yes’ (2); all others were classified as ‘No’ (1).

Further work-related covariates were paid weekly working hours and night shift work. Paid weekly working hours (h) were determined using the following question: ‘How many hours do you normally work in total per week? Please only enter the paid hours (h).’ Weekly working hours were grouped into six categories: <20 h (1), 20 h–<35 h (2), 35 h–<41 h (3), 41 h–<49 h (4), 49 h–<55 h (5), and ≥ 55 h (6). These categories are based on Dragano et al. [54], with additional adjustments made for the first category (< 20 h). In the study, night shift work was categorized as either ‘No’ (1) or ‘Yes’ (2). The ‘Yes’ category included the subcategories ‘Constantly every working day’, ‘Regularly, but not every working day’, and ‘Irregularly, occasionally on some working days’.

### Statistical analysis

Firstly, a descriptive analysis was performed to provide an overview of SP. Characteristics of the study population were summarized as absolute frequencies and percentages, for continuous factors, giving mean and standard deviation (SD). Secondly, prevalence ratios (PRs) with 95% confidence intervals (CIs) were calculated using robust Poisson regressions to assess the association between occupational segments (reference: all other occupations combined) and SP, as well as between managerial/ supervisory status (reference: non-supervisors/non-managers) and SP. In additional analyses, managers/supervisors were compared with non-managers/non-supervisors within each occupational segment. We included the missing values of the confounders as separate categories in the analysis for maintaining sample size. Missing values ranged from 0% to 0.9% across variables (see Table 1 for details). Four adjustment models were applied as follows:

- Model 1 (M1): adjusted for age (continuous variable), age**2 (square of continuous variable), and sex.
- Model 2 (M2) – main model: M1, additionally, educational level, and health status.
- Model 3 (M3): M2, additionally, migration background, relative income, marital status, and the presence of children.
- Model 4 (M4): M2, additionally, paid working hours, and night shift work.

Models 3 and 4 include variables that may not only act as true confounders but may also mediate the effect of occupation on SP. For example, having children may influence occupational career and therefore act as a confounder. At the same time, occupation may also affect the likelihood of having children, in which case the variable may mediate the association between occupation and SP. For this reason, Model 2 was selected as the main model. Models 3 and 4 may nevertheless contribute to a better understanding of the mechanisms leading from occupation to SP. In the ‘health status’ variable, the categories ‘Less good’ (4) and ‘Poor’ (5) were combined in the analysis to reduce model complexity, as both categories indicate an unfavorable health status. Models 1 to 4 were used to analyze the relationship between occupational segments and SP. To assess whether the association between occupational segment and SP differed by supervisory status, the analysis was conducted using a robust Poisson regression model that included an interaction term between occupational segment and supervisory or managerial status. Marginal effects from this model were used to estimate prevalence ratios and 95% confidence intervals for each occupational segment separately among employees with and without managerial or supervisory responsibilities. Within each stratum (supervisors/managers and non-supervisors/non-managers), the SP risk in each occupational segment was compared with the combined group of all other segments, which served as the reference category. The interaction term was analyzed to formally test whether the association between occupational segments and SP differed between employees with and without supervisory roles. All statistical analyses were conducted using R version 4.2.2.

## Results

### Descriptive summary

Of the 86,855 employees who were ill at least once in the past 12 months in such a way that they were unable to pursue their usual professional activities, 76.4% reported having worked while ill at least once in the past 12 months. More specifically, 35.7% of participants went to work once during illness in the last 12 months, while 34.7% reported going to work two to five times during illness. Furthermore, 6.0% of participants went to work more than five times in the past 12 months despite being ill. About 23.6% did not report any SP in the last year.

Table 1 summarizes the sociodemographic and work-related characteristics of the study population according to sickness presenteeism (SP) status. Overall, SP was more common among women than men. Participants reporting SP also tended to report poorer self-rated health, longer paid working hours, and night shift work compared with those who did not report SP. In contrast, SP prevalence was broadly similar across age groups, educational levels, migration background, income groups, and marital status. Only small differences were observed according to the presence of children under 14 years in the household. Detailed descriptive statistics are presented in Table 1.

**Table 1:** Description of study population based on the reporting of SP

Age	All employees (*N* = 86,855)	SP yes (*N* = 66,370)	SP no (*N* = 20,485)
	45.7 ± 10.5	45.5 ± 10.4	46.3 ± 10.7
**Sex**			
Men	41,987	30,836 (73.4%)	11,151 (26.6%)
Women	44,868	35,534 (79.2%)	9,334 (20.8%)
**Educational level**			
Low	1,150	857 (74.5%)	293 (25.5%)
Moderate	35,651	27,422 (76.9%)	8,229 (23.1%)
High	50,031	38,072 (76.1%)	11,959 (23.9%)
Missing value	23	19 (82.6%)	4 (17.4%)
**Health status**			
Excellent	3,035	1,945 (64.1%)	1,090 (35.9%)
Very good	28,619	20,815 (72.7%)	7,804 (27.3%)
Good	47,696	37,345 (78.3%)	10,351 (21.7%)
Less good	7,020	5,869 (83.6%)	1,151 (16.4%)
Poor	364	296 (81.3%)	68 (18.7%)
Missing value	121	100 (82.6%)	21 (17.4%)
**Migration background**			
No	75,149	57,367 (76.3%)	17,782 (23.7%)
Yes	11,694	8,994 (76.9%)	2,700 (23.1%)
Missing value	12	9 (75.0%)	3 (25.0%)
**Income**			
<60%	5,989	4,531 (75.7%)	1,458 (24.3%)
60%–<150%	58,651	45,106 (76.9%)	13,545 (23.1%)
≥150%	18,425	13,932 (75.6%)	4,493 (24.4%)
Missing value	3,790	2,801 (73.9%)	989 (26.1%)
**Marital status**			
Single	26,777	20,550 (76.7%)	6,227 (23.3%)
Married living together	48,631	36,951 (76.0%)	11,680 (24.0%)
Married living apart	1,557	1,211 (77.8%)	346 (22.2%)
Divorced	8,752	6,809 (77.8%)	1,943 (22.2%)
Widowed	1,122	836 (74.5%)	286 (25.5%)
Missing value	16	13 (81.3%)	3 (18.8%)
**Presence of children under 14 years**			
No	63,355	48,110 (75.9%)	15,245 (24.1%)
Yes	23,482	18,252 (77.7%)	5,230 (22.3%)
Missing value	18	8 (44.4%)	10 (55.6%)
**Paid working time**			
<20	5,412	3,829 (70.8%)	1,583 (29.2%)
20–34	18,423	14,244 (77.3%)	4,179 (22.7%)
35–40	52,701	40,067 (76.0%)	12,634 (24.0%)
41–48	6,320	4,983 (78.8%)	1,337 (21.2%)
49–54	1,967	1,642 (83.5%)	325 (16.5%)
≥55	1,251	1,068 (85.4%)	183 (14.6%)
Missing value	781	537 (68.8%)	244 (31.2%)
**Night shift work**			
No	71,248	53,609 (75.2%)	17,639 (24.8%)
Yes	15,607	12,761 (81.8%)	2,846 (18.2%)

Values in column ‘All employees’ show absolute numbers or mean and standard deviation for continuous variables

Values in column ‘SP yes’ and ‘SP no’ show absolute numbers (row percentage) or mean and standard deviation for continuous variables

### Results on SP prevalence in occupational segments

**Fig. 2:**
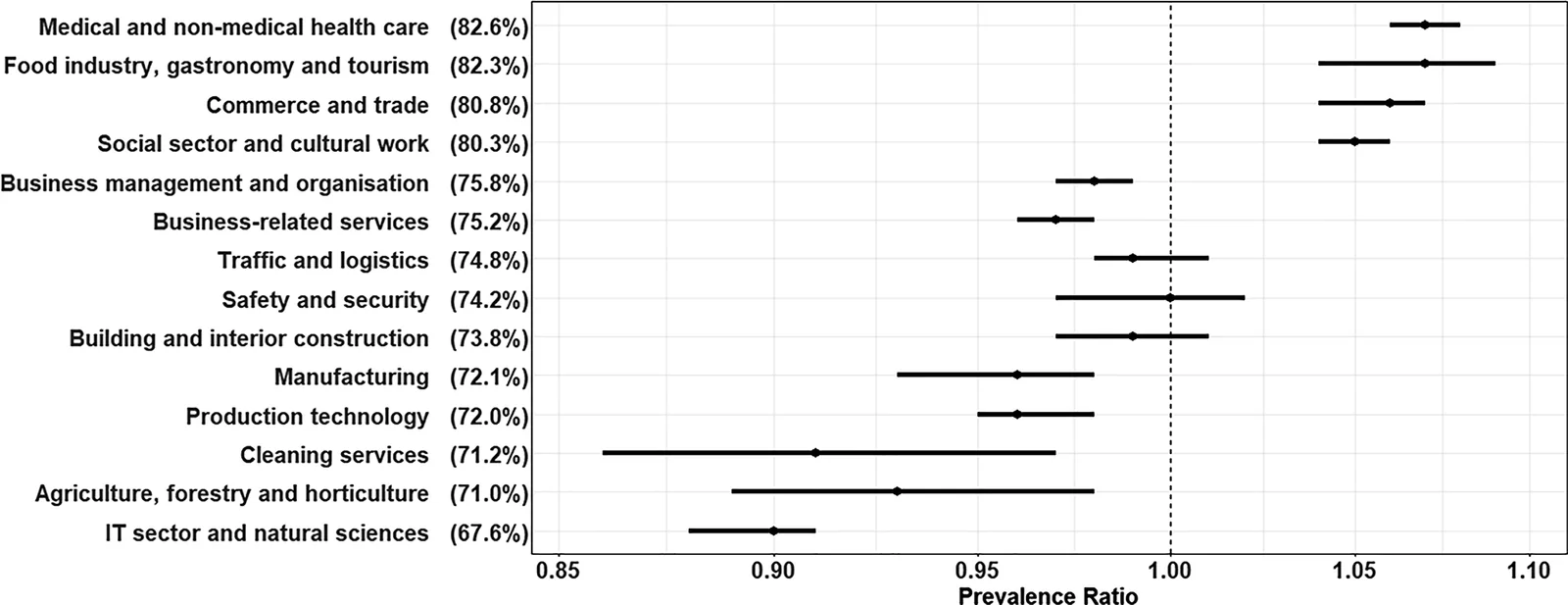
Prevalence ratios (PRs) and 95% confidence intervals (CIs) for sickness presenteeism (SP) by occupational segments from robust Poisson regression (Model 2). Each occupational segment was compared with all other occupations combined as the reference group. Within each stratum, the SP risk in each occupational segment was compared with the combined group of all other segments, which served as the reference category. The dashed vertical line at PR = 1.00 indicates no difference between a segment and all others. PRs were estimated only for segments with at least 10 cases per stratum. Models were adjusted for age, sex, education, and health status. SP prevalences for each occupational segment are presented in parentheses

Figure 2 shows the prevalence and prevalence ratios of SP across occupational segments. Segments with the highest prevalence of SP were found in health care (82.6%), food, gastronomy and tourism (82.3%), commerce and trade (80.8%), and social/cultural services (80.3%), while business, logistics, safety, and construction occupations showed moderate prevalence (73.8–75.8%). Lower prevalence was observed in manufacturing, production technology, cleaning, and agriculture (71–72%), with the lowest in IT and natural sciences (67.6%).

The analysis of prevalence ratios (PRs) across occupational segments revealed that employees in medical and non-medical healthcare occupations (PR = 1.07; 95% CI = 1.06–1.08), as well as those in the food, gastronomy, and tourism sectors (PR = 1.07; 95% CI = 1.04–1.09), showed higher PRs, indicating a higher risk of SP compared to the reference. Similarly, occupations in commerce and trade (PR = 1.06; 95% CI = 1.04–1.07) and service occupations in the social and cultural sectors (PR = 1.05; 95% CI = 1.04–1.06) also displayed elevated PRs, though to a lesser extent. In contrast, most other occupational segments, including business-related service occupations, traffic and logistics, safety and security, construction, manufacturing, production technology, cleaning, and agriculture, had PRs below or close to 1, suggesting no substantial increase in risk relative to the reference. The lowest SP prevalence was observed in employees in the IT sector and natural sciences (67.6%), who showed a significantly lower risk of SP compared with all other occupational segments (PR = 0.90; 95% CI = 0.89–0.92). Results for Model 2 are presented in Fig. 2, while the estimates for Models 1–4 are provided in Additional file 2.

The prevalence ratios did not substantially differ between Models 1–4, indicating that SP prevalence ratios in single occupational segments are rather independent of level of education, health status, migration background, income, marital status, presence of children, paid working time, and night shift work. The results of all 4 models are shown in Additional file 2.

To further explore occupation-specific differences, supplementary analyses were conducted at the 3-digit occupational group level. The corresponding prevalence estimates and prevalence ratios for all four models are presented in Additional file 3. Overall, the findings support the SP pattern observed in occupational segments. The prevalence ratios did not substantially differ across Models 1–4. Within the healthcare sector, statistically significantly higher SP risk was observed among veterinary medicine and non-medical health practitioners (PR = 1.20; 95% CI = 1.14–1.27, Model 2), professionals in human medicine and dentistry (PR = 1.17; 95% CI = 1.15–1.20), and workers in nursing, emergency medical services, and obstetrics (PR = 1.08; 95% CI = 1.06–1.09). In the food, gastronomy, and tourism sectors, elevated PRs were found for beverage production (PR = 1.23; 95% CI = 1.11–1.37), gastronomy (PR = 1.08; 95% CI = 1.04–1.12), and cooking occupations (PR = 1.07; 95% CI = 1.03–1.11). In the social and cultural sectors, statistically significant elevated SP risks were observed in occupations in stage, costume and prop design (PR = 1.24; 95% CI = 1.14–1.36), in musicians, singers and conductors (PR = 1.14; 95% CI = 1.07–1.21), and in teachers in general education (PR = 1.10; 95% CI = 1.09–1.12). Similarly, within commerce and trade, elevated SP risk was found for sales occupations in retail trade (PR = 1.08; 95% CI = 1.06–1.10) and sales occupations (retail trade) selling durable goods involving clothing, electronic devices, and furniture (PR = 1.08; 95% CI = 1.03–1.13). In contrast, occupations in mathematics and statistics (PR = 0.84; 95% CI = 0.73–0.97) and software development and programming (PR = 0.80; 95% CI = 0.76–0.84) showed the lowest SP risks (Additional file 3).

### Results on SP prevalence in occupational segments concerning the hierarchical position of employees

Compared to employees without supervisory or managerial responsibilities, those in supervisory or managerial positions had a higher risk of working while sick (PR = 1.08; 95% CI = 1.07–1.10, Table 2). The prevalence ratios did not substantially differ between Models 1–4. Sex-stratified analyses showed an elevated risk for both men and women; however, the association was much more pronounced among men than among women (men: PR = 1.11; 95% CI = 1.09–1.12; women: PR = 1.04; 95% CI = 1.02–1.06; Table 2).

**Table 2:** Prevalence ratios (PRs) and 95% confidence intervals (CIs) of the hierarchical position of employees on sickness presenteeism (SP)

Variable	Category	N	SP prevalence	Model 1 PR (95% CI)	Model 2 (main model) PR (95% CI)	Model 3 PR (95% CI)	Model 4 PR (95% CI)
				(adjusted for age, sex)	(adjusted for Model 1, educational level, health status)	(adjusted for Model 2, migration background, income, marital status, the presence of children)	(adjusted for Model 2, paid working time, night shift work)
Supervisors and managers	No	78,066	76.1%	1 ---	1 ---	1 ---	1 ---
	Yes	6,664	80.7%	1.08 (1.07–1.09)	1.08 (1.07–1.10)	1.08 (1.07–1.09)	1.07 (1.06–1.09)
	Missing values	2,125					
	Total	86,855					
*Females* *only*	No	41,668		1 ---	1 ---	1 ---	1 ---
	Yes	2,177		1.03 (1.01–1.06)	1.04 (1.02–1.06	1.04 (1.02–1.06)	1.02 (1.00–1.05)
	Missing values	1,023					
	Total	44,868					
*Males only*	No	36,398		1 ---	1 ---	1 ---	1 ---
	Yes	4,487		1.10 (1.09–1.12)	1.11 (1.09–1.12	1.10 (1.09–1.12)	1.10 (1.08–1.12)
	Missing values	1,102					
	Total	41,987					

**Fig. 3:**
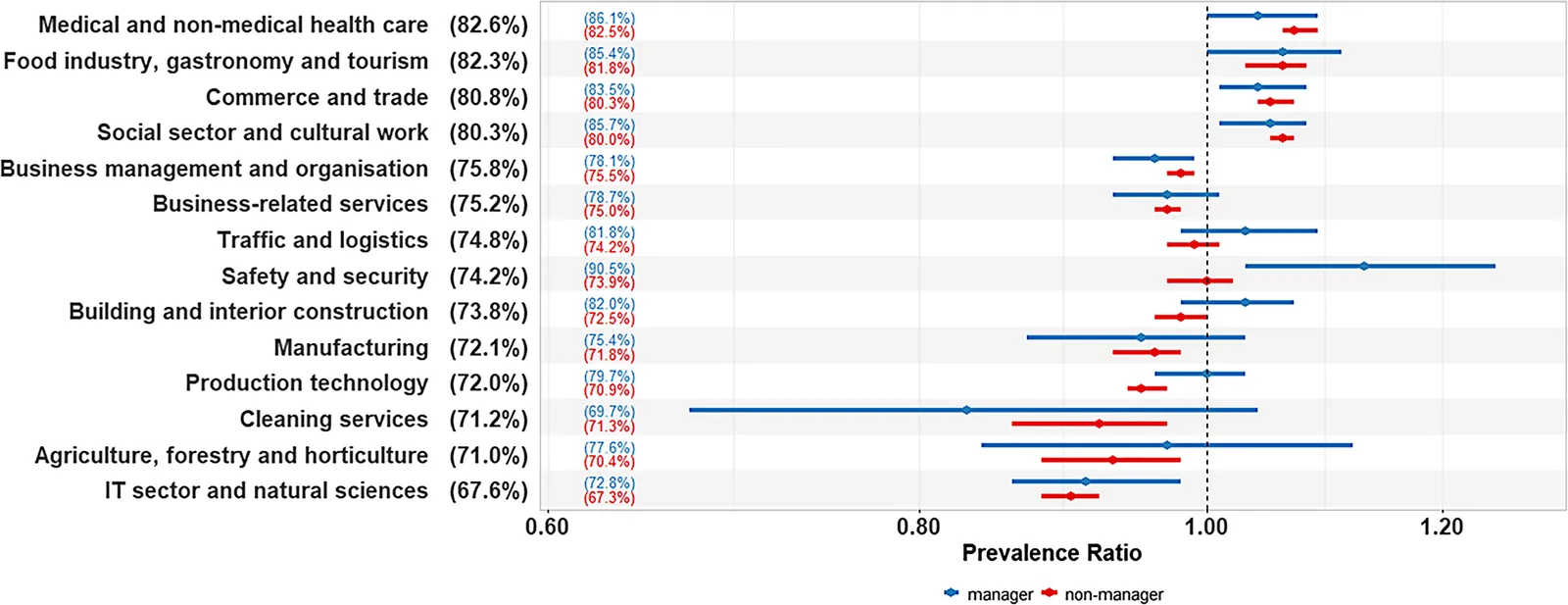
Prevalence ratios and 95% confidence intervals for sickness presenteeism (SP) across occupational segments by supervisory/managerial status (Model 2). Points show adjusted prevalence ratios (PRs) with 95% confidence intervals from robust Poisson regression; blue = supervisors/managers, red = non-supervisors/non-managers. Within each stratum, the SP risk in each occupational segment was compared with the combined group of all other segments, which served as the reference category. The dashed vertical line at PR = 1.00 indicates no difference between a segment and all others. Percentages in parentheses give SP prevalence (black = overall, blue/red = stratum-specific). PRs were estimated only for segments with at least 10 cases per stratum. Models were adjusted for age, sex, education and health status

Figure 3 shows the prevalence of SP and prevalence ratios in different occupational segments with regard to the hierarchical position of employees (i.e., supervisory/managerial versus non-supervisory/non-managerial positions) for the main model (Model 2). Overall, the results indicate that SP varies primarily across occupational segments rather than between managers and non-managers. Both managers and non-managers showed statistically significant elevated prevalence ratios in occupations with generally higher SP prevalence, namely healthcare occupations (manager: PR = 1.04 and non-manager: PR = 1.07), food industry, gastronomy and tourism (manager: PR = 1.06 and non-manager: PR = 1.06), commerce and trade (manager: PR = 1.04 and non-manager: PR = 1.05), and service occupations within the social and cultural sector (manager: PR = 1.05 and non-manager: PR = 1.06). In addition, only in safety and security occupations did managers exhibit a statistically higher prevalence ratio (PR = 1.13; 95% CI: 1.03–1.25) compared to non-managers (PR = 1.00; 95% CI: 0.97–1.02). Furthermore, a statistically significant difference between managers and non-managers was observed in building and interior construction. The PR among managers was not itself significantly elevated (PR = 1.03; 95% CI: 0.98–1.07), but the PR among non-managers was slightly below unity (PR = 0.98; 95% CI: 0.96–1.00). A similar pattern was observed for occupations concerned with production technology, with a lower PR among non-managers (PR = 0.95; 95% CI: 0.94–0.97), whereas the PR among managers was close to the null (PR = 1.00; 95% CI: 0.96–1.03). In contrast, no statistically significant difference between managers and non-managers was observed in traffic and logistics occupations. Results from all four models (Models 1–4) are presented in Additional file 4.

## Discussion

This study shows differences in SP prevalence across occupational segments based on baseline data from the NAKO cohort. The highest prevalence was observed in healthcare, the food industry and hospitality sector, commerce and trade, and social and cultural occupations. By contrast, a lower prevalence of SP was found in more technical and manufacturing-related sectors. The additional analysis at the occupational-group level revealed substantial heterogeneity within these broad segments, suggesting that specific occupational characteristics may provide additional insights into SP beyond those captured by broad sectoral classifications alone. Supervisors/managers showed a slightly higher overall SP prevalence than non-supervisors/non-managers, with a stronger association among men (PR = 1.11; 95% CI = 1.09–1.12) than among women (PR = 1.04; 95% CI = 1.02–1.06). However, occupation-specific analyses showed no consistent pattern in the differences between supervisors/managers and non-supervisors/non-managers, suggesting that occupational context may be more important for SP than hierarchical position alone.

The overall prevalence of SP was 76.4% in this study. This result is broadly consistent with the findings from previous studies using comparable definitions and timeframes, including SP prevalences of 72.7% in Denmark [17] and 70.1% in Sweden [41]. However, direct comparisons of SP prevalence across studies remain challenging because there is no standardized approach for defining and measuring SP. Studies differ not only in the populations included but also in the operational definition of SP, the threshold used to classify presenteeism, the recall period, and the wording of survey questions [4, 21, 55].

An important methodological difference concerns the definition of the study population. While some studies estimate SP among all employees, others restrict analyses to employees who reported a health problem during the recall period. Because employees without illness cannot exhibit SP by definition, including them generally leads to substantially lower prevalence estimates. In this study, SP prevalence would have been approximately 10% points lower (66.1%) when employees without recent illness had been included, which highlights the importance of sample definition. This finding is consistent with Navarro et al. (2018), who reported substantial differences between sick employees (65.3%) and all employees (28.3%).

Studies from Germany have similarly reported considerably lower estimates than the present study, including 27.9% [56], 34.6% [57] and 57% [6]. These differences may reflect variations in the operationalization of SP. For example, d’Errico et al. [57] required employees to have worked while ill for at least two days, whereas the present study classified employees as having SP if they had worked while ill at least once during the previous 12 months. The estimate reported by Lohmann-Haislah [6], in contrast, combined employees reporting SP only with those reporting both SP and sickness absence. Thus, differences in eligibility criteria and thresholds can substantially affect prevalence estimates [21, 55, 58]. In addition to methodological variation, SP prevalence may also change over time [8], for example, a recent longitudinal study from South Korea demonstrated a decline in SP during and after the SARS-CoV-2 pandemic, illustrating that broader societal and workplace changes can influence attendance behavior [9]. Despite similar recall periods and definitions, international estimates of SP vary considerably, ranging from 19.7% to 63.0% across Organization for Economic Co-operation and Development (OECD) countries [56] and averaging 43.6% across 12 European countries [43]. Thus, the comparatively high prevalence observed in the present study is likely explained by a combination of methodological differences, population characteristics, temporal developments, and contextual factors rather than by a single underlying level of sickness presenteeism.

The objective of this study was to investigate the association between occupational segments and the prevalence of SP. The results indicate substantial variation in SP prevalence across occupational segments, with the highest prevalence observed in occupations related to healthcare, food and catering, trade, and social and cultural services. These occupations are typically characterized by high job demands, strong attendance pressure, and limited possibilities for replacement during absence. Several factors may explain the elevated SP prevalence in these sectors. Healthcare and related service occupations are often associated with high workloads, considerable job stress, and long working hours [8], all of which have been shown to be positively associated with SP [21]. In addition, employees frequently experience a strong sense of professional responsibility and commitment toward patients, clients, or children, which may encourage attendance despite illness [8, 21]. Staffing shortages, specialized job requirements, and difficulties in finding suitable replacements further increase attendance pressure and concerns about burdening colleagues [13, 25, 41, 59, 60]. Similar mechanisms may operate in hospitality, gastronomy, tourism, and food service occupations, which are characterized by intensive customer interaction, inflexible scheduling, and low staffing redundancy. In these settings, employee absence can immediately disrupt service delivery and business operations, creating strong pressure to attend work despite illness, particularly in more precarious employment contexts [55, 58, 61]. Elevated levels of SP have also been reported in commerce and trade, where high workloads may be combined with comparatively low wages [30]. Frequent customer contact may make absence particularly visible, while lean staffing structures can increase workloads for colleagues when an employee is absent [5, 17, 55].

The additional analysis at the occupational-group level provides further support for this interpretation. Elevated prevalence ratios were observed for stage, costume and prop design (PR = 1.24), beverage production (PR = 1.23), veterinary medicine and non-medical animal health (PR = 1.20), human medicine and dentistry (PR = 1.17), musicians, singers and conductors (PR = 1.14), teaching in general education schools (PR = 1.10), nursing, emergency medical services and obstetrics (PR = 1.08), sales occupations in retail trade (PR = 1.08), gastronomy (PR = 1.08), cooking occupations (PR = 1.07), and education and social work (PR = 1.05). The findings suggest that occupational differences in SP are not solely attributable to broad sectoral membership but may also reflect characteristics specific to particular occupations and work tasks. The elevated prevalence observed across a diverse range of occupations may indicate that occupational contexts may shape the likelihood of working while ill through a combination of physical, psychosocial, organizational, and task-specific demands.

Thus, findings can be further interpreted in light of occupation-specific indicators of physical and environmental demands, work intensity, working-time arrangements, and autonomy, which are provided by the BAuA Job-Exposure Matrix (JEM) [62–64]. The elevated SP observed in healthcare, education, gastronomy, and retail occupations is therefore compatible with occupational profiles characterized by comparatively high work intensity, substantial interpersonal demands, restricted flexibility in working-time arrangements, and, for some occupations, considerable physical demands. Previous studies similarly indicate that workload, low replaceability, and social or professional responsibility are important determinants of SP [21, 30, 55, 58]. Importantly, these mechanisms correspond to several dimensions captured by the BAuA-JEM, particularly work intensity, working-time constraints, and limited autonomy, and are also consistent with occupational stress models emphasizing the interaction between job demands and job resources [65, 66]. Thus, physical demands, work intensity, environmental demands, working-time constraints, autonomy, and replaceability may interact in occupation-specific ways. Accordingly, occupational differences in SP may depend not only on the level of job demands but also on the extent to which employees have sufficient autonomy and flexibility to adjust their work when experiencing health problems.

Conversely, the occupational-group analysis identified some occupational groups with lower SP, particularly in information-, technology-, mathematics-, and science-related occupations. The lowest PRs among occupational groups with sufficiently large numbers of cases were observed for software development and programming (PR = 0.80), mathematics and statistics (PR = 0.84), IT system analysis, IT application consulting and IT sales (PR = 0.89), as well as technical research and development (PR = 0.93). Previous research suggests that occupations primarily involving the handling of information, symbols, or data generally exhibit lower levels of SP than occupations centered on direct interactions with patients, customers, or clients [41]. This finding suggests that lower SP may partly reflect the availability of job resources and flexibility rather than simply lower occupational strain. Many information- and knowledge-intensive occupations offer greater autonomy over the sequencing and timing of tasks, greater opportunities to work independently, and, increasingly, opportunities for remote work. These characteristics may reduce the immediate attendance pressure associated with illness. Thus, higher occupational autonomy and flexible working-time arrangements may allow employees to adjust the timing or intensity of work according to their health status. At the same time, remote work does not necessarily eliminate SP; rather, it may change its form by enabling employees to continue working while ill from home. Thus, greater flexibility may reduce conventional workplace SP while potentially increasing so-called tele-SP [67, 68]. For future research, it would be interesting to move beyond broad occupational classifications and examine the specific working conditions through which occupational characteristics influence decisions to work while ill.

The observed variation in SP prevalence across occupations is consistent with previous research reporting higher levels of SP in healthcare, education, commerce, and hospitality occupations [18, 21, 30, 57, 69]. Adjustment for sex and age, educational level, health status, migration background, income, marital status, presence of children, working hours, and night-shift work did not substantially alter the association between occupational segment and SP in the present study. However, the difference between crude SP prevalence and adjusted PRs was more pronounced for business-related services and business management and organization. These occupational segments had a comparatively high proportion of women (62.8% in business-related services and 65.8% in business management and organization), and adjustment for sex might have attenuated the associations despite their relatively high crude SP prevalence. This may suggest that differences in the sex composition of occupational segments may partly contribute to the apparent discrepancy between crude prevalence and adjusted estimates. Nevertheless, the persistence of occupational differences after adjustment indicates that these differences are not fully explained by sex or the other measured sociodemographic, socioeconomic, health-related, and work-related characteristics. Further factors specific to occupational tasks, working conditions, and organizational contexts may therefore contribute to differences in SP across occupational segments.

Although many of the observed associations were statistically significant, their magnitude varied considerably and was generally moderate. For example, prevalence ratios of 1.05–1.08 correspond to approximately 5–8% higher prevalence of SP relative to the reference group. These differences should therefore be interpreted not only in terms of statistical significance but also in relation to their absolute and population-level implications. Given the high overall prevalence of SP in the study population (76.4%), even moderate relative differences may translate into a considerable number of additional employees working while ill, particularly in large occupational groups. At the same time, the magnitude of most associations does not suggest that occupational group alone is a strong determinant of SP. Rather, the findings indicate systematic differences in the likelihood of working while ill that may reflect the cumulative influence of occupational working conditions, organizational factors, and individual characteristics.

To our knowledge, this is the first study to compare SP prevalence between employees with and without supervisory or managerial responsibilities across a broad range of occupations in Germany. Consistent with previous findings [18, 21], supervisors and managers exhibited a higher overall prevalence of SP than employees without leadership responsibilities. This elevated prevalence may be attributable to the structural and psychosocial demands associated with leadership positions. Managerial roles are often characterized by high workloads, extensive responsibility, and increased psychosocial strain [66, 70, 71], factors that have been linked to adverse health outcomes and a greater likelihood of working despite illness. In particular, managers frequently face role conflict, persistent time pressure, and substantial emotional demands, such as supporting employees while simultaneously meeting organizational targets. These pressures may foster a perceived obligation to remain present at work even when experiencing health problems.

Sex-stratified analyses further showed that supervisory or managerial responsibilities were associated with a higher prevalence of SP in both women and men, although the association was more pronounced among men (PR = 1.11) than among women (PR = 1.04). This finding suggests that the relationship between leadership responsibilities and SP may differ by sex, potentially reflecting differences in the reasons for working while ill. In a study among Swedish general practitioners, women more frequently reported working despite illness because of concern for patients and colleagues, high workload, and difficulties finding replacements, whereas men more often reported feeling sufficiently healthy to work or concerns about the financial consequences of sickness absence [72]. Similarly, Luksyte et al. [73] found that sick men were more likely to prioritize protecting job performance and engage in extra-role behaviors despite illness, whereas women were more likely to prioritize health preservation. Thus, determinants of SP may differ between women and men, even when overall prevalence is similar.

The stronger association between supervisory responsibilities and SP observed among men may reflect differences in the leadership roles occupied by men and women. Men remain overrepresented in senior management positions, which are typically associated with broader organizational responsibility, longer working hours, and stronger expectations of continuous availability [74]. In contrast, women in managerial positions are more frequently employed in healthcare, education, and public administration; occupational sectors that already exhibit comparatively high levels of SP irrespective of leadership status [69]. Consequently, the additional association between supervisory responsibilities and SP may be less pronounced among women because attendance pressure related to the underlying occupation is already substantial.

Furthermore, although employees with supervisory or managerial responsibilities exhibited a higher overall prevalence of SP, the difference between with and without such responsibilities was not clearly apparent across many occupational segments. Estimates for employees with supervisory or managerial responsibilities were less precise in several segments, as reflected by wider confidence intervals, likely due to the smaller number of participants in these subgroups. This limited precision may also increase the potential for model instability, particularly given the number of covariates included in the Poisson regression models. However, the estimates were highly consistent across all four model specifications, with no substantial changes in prevalence ratios or their overall pattern following progressive adjustment. In addition, supplementary analyses based on crude models yielded very similar results. This consistency provides reassurance regarding the robustness of the findings. Overall, the broadly similar patterns observed across occupational segments suggest that occupational context may exert a stronger and more consistent influence on SP than hierarchical position alone. In healthcare, for example, high SP among non-supervisory employees may primarily reflect direct patient contact, limited replacement opportunities, staffing shortages, and professional responsibility, while managers may additionally face leadership and staffing-related pressures [8, 21, 59, 69]. Similarly, the comparatively high SP observed among managers in safety and security occupations may reflect strong expectations of availability, limited replaceability, role-model expectations, and responsibility in critical situations [25, 75, 76].

In addition, the largest differences between employees with and without supervisory or managerial responsibilities were observed in building and interior construction and production technology. In both segments, the differences were primarily driven by comparatively lower SP among employees without supervisory or managerial responsibilities (PR = 0.98, 95% CI 0.96–1.00, and PR = 0.95, 95% CI 0.94–0.97, respectively), whereas the estimates for employees with supervisory or managerial responsibilities were close to the null in production technology (PR = 1.00, 95% CI 0.96–1.03) and slightly elevated in building and interior construction (PR = 1.03, 95% CI 0.98–1.07). The differences may reflect differences in the organization of work within these occupational segments rather than differences in the overall occupational demands of the sectors. Employees with supervisory or managerial responsibilities may face greater time pressure, responsibility for workflow coordination, and expectations of availability, factors previously associated with SP [77]. Differences in staffing, replaceability, team responsibilities, and workplace attendance norms may further contribute to the observed pattern [30, 55]. Leadership behavior may further influence SP by shaping SP norms and attendance expectations among employees [78, 79]. Overall, the findings suggest that the relationship between hierarchical position and SP may depend on the specific organization of work within occupational segments.

Taken together, our findings suggest that SP reflects a complex interplay of occupational characteristics, leadership responsibilities, and sex. The higher overall SP observed among supervisors and managers indicates that leadership responsibilities may represent an additional factor associated with SP. However, the heterogeneous patterns across occupational segments suggest that this association is strongly embedded in the broader occupational context. Factors such as work intensity, autonomy, working-time constraints, interpersonal demands, and replaceability may help explain why SP varies substantially between occupations. Future research should therefore examine SP together with specific occupational and organizational working conditions and consider sex-specific mechanisms underlying the decision to work while ill.

### Strengths and limitations

This study has several limitations that should be considered when interpreting the findings. First, the cross-sectional design precludes conclusions about temporal relationships between occupational characteristics and SP. Longitudinal studies are therefore needed to examine SP over time. Second, information on SP was collected via self-reports from participants regarding their SP behaviors over the past 12 months. This may have introduced recall and social desirability bias. Although alternative assessments by supervisors or coworkers could be considered, such reports may also be inaccurate because health problems and SP are not always observable to others [4]. Moreover, the absence of standardized SP measures limits comparability across studies, including differences in recall periods, response scales, and criteria used to define SP [42, 80]. In addition, the measurement approach used in the present study did not allow calculation of SP propensity, which has been proposed as a more informative indicator because it reflects the likelihood of choosing SP over sickness absence when ill [4, 18, 81]. Furthermore, SP was assessed as the number of occasions on which employees attended work despite illness rather than the number of days worked while ill. Consequently, the measure captures the occurrence but not the duration of SP episodes, and employees with both short and prolonged periods of working while ill may have been classified into the same response category. Future studies should complement episode-based measures with information on the duration of sickness presenteeism (i.e., the number of days worked while ill) or assess presenteeism propensity, as these measures better capture the intensity of sickness presenteeism and employees’ attendance decisions when ill [5, 81, 82].

Several limitations are also related to the NAKO study design. Data collection occurred over a five-year period (from March 2014 to September 2019), during which changes in participants’ personal and occupational circumstances may have influenced the results [53]. It should also be noted that the data were collected before the SARS-CoV-2 pandemic. Therefore, the observed prevalence estimates may not fully reflect current working conditions, which have been shaped by changes in workplace policies and other pandemic-related developments [83]. These changes have influenced employee workplace behavior, including the tendency to work from home [84], which may increase SP prevalence [85, 86]. At the same time, recent evidence suggests that SP prevalence may have declined following the pandemic [9]. Replication using more recent data is therefore warranted.

Selection bias may further limit generalizability. The NAKO cohort had a relatively low response of 15.6% and was not fully representative of the German population, with middle-aged individuals, persons with higher educational attainment, and individuals without a migration background being slightly overrepresented [35]. These differences may be relevant because age, education, migration background, occupational position, and working conditions are associated with SP. In particular, the overrepresentation of highly educated participants may have resulted in a greater representation of professional, highly qualified, and managerial occupations and could contribute to an overestimation of overall SP prevalence, as higher educational attainment has been associated with a greater likelihood of SP in previous studies [13, 68]. Nevertheless, adjustment for age, educational level, migration background, health status, socioeconomic characteristics, and relevant work-related factors did not substantially change the associations between occupational segment and SP, suggesting that these characteristics are unlikely to fully account for the observed occupational differences. Selection bias may also be reflected in the unequal distribution of occupational segments, partly due to the overrepresentation of highly educated participants and of those without a migration background. The large representation of social and cultural work and healthcare may have contributed to the comparatively high overall SP prevalence observed in this study compared to other studies in Germany. In contrast, the smaller number of participants in occupations such as cleaning services and agriculture, forestry and horticulture may have affected the precision of corresponding PR estimates.

In addition, the distinction between the response options “No, never” and “Does not apply” in the NAKO questionnaire was not explicitly defined and could not be differentiated in the available dataset, which may have resulted in varying interpretations among participants (Romero Starke et al., 2021, unpublished report). However, because participants were excluded from the analytical sample only if they additionally reported no days of illness impairing their work during the previous 12 months, we expect any resulting misclassification to have had only a limited impact on the observed associations.

Despite these limitations, the study has several important strengths. Its large sample size enabled robust prevalence estimates and detailed analyses across occupational segments. Furthermore, occupations were classified using the standardized and hierarchically structured German Classification of Occupations (KldB 2010) [36], allowing for systematic comparisons across occupational groups. Finally, the examination of SP prevalence across occupational segments, respectively occupational groups and by supervisory or managerial responsibility provides novel insights into occupational differences in SP and contributes to a better understanding of occupation-specific risk patterns in the workforce.

## Conclusions

This study demonstrates that sickness presenteeism is highly prevalent among employees in Germany and varies substantially across occupational contexts. Although many occupational differences were moderate in relative terms, their potential population-level relevance should not be underestimated given the high overall prevalence of SP. The highest prevalence was observed in healthcare, food and hospitality, commerce and trade, and social and cultural occupations, where high job demands, strong responsibilities toward patients, clients, or customers, and limited opportunities for replacement may encourage employees to work despite illness. In contrast, particularly low prevalence was observed in IT-, mathematics-, and science-related occupations, where greater autonomy, flexible work organization, and opportunities to work remotely may provide employees with greater flexibility to adjust the timing and organization of their work when experiencing health problems. Although supervisors and managers showed slightly higher overall prevalence of SP than employees without leadership responsibilities, occupation-specific analyses suggest that occupational segment is a more important determinant of SP than hierarchical position alone. The stronger association between supervisory responsibilities and SP among men further indicates that the relationship between leadership and SP may vary by sex.

Overall, these findings underscore the complex nature of SP, which is shaped by a combination of occupational demands, organizational structures, and workplace cultures, and demographic characteristics. Accordingly, efforts to reduce SP should focus on occupations with particularly high prevalence and address the underlying conditions that encourage attendance despite ill health.

## Supplementary Information

Below is the link to the electronic supplementary material.

Supplementary Material 1

Supplementary Material 2

Supplementary Material 3

Supplementary Material 4

## Data Availability

This project was conducted with data (Application No. NAKO-888) from the German National Cohort (NAKO) (www.nako.de). Access to and use of NAKO data can be obtained via an electronic application portal (https://transfer.nako.de).

## Abbreviations

CI: Confidence interval
h: Hour
ISCED 97: International Standard Classification of Education 1997
KldB: German Classification of Occupations
M1: Model 1
M2: Model 2
M3: Model 3
M4: Model 4
NAKO: German National Cohort (Nationale Kohorte)
OECD: Organization for Economic Co-operation and Development
PR: Prevalence ratio
SD: Standard deviation
SP: Sickness presenteeism
