# Supplementary material for: Occupational differences in sickness presenteeism in the baseline survey of the German National Cohort (NAKO)
Source: J Occup Med Toxicol. 2026 Sep 25;21:31. doi: 10.1186/s12995-026-00529-1 (PMC13615606; doi:10.1186/s12995-026-00529-1)
Supplement: Supplementary file 1 [file 12995_2026_529_MOESM1_ESM.docx]

**Additional file 1:** Overview of occupational segments and occupational groups

| **Occupational segment** | **Main occupational group** | **Occupational group** |
| --- | --- | --- |
| S11 Occupations in agriculture, forestry and horticulture | 11 Occupations in agriculture, forestry, and farming | 111 Occupations in farming |
|  |  | 112 Occupations in animal husbandry |
|  |  | 113 Occupations in horsekeeping |
|  |  | 114 Occupations in fishing |
|  |  | 115 Occupations in animal care |
|  |  | 116 Occupations in vini- and viticulture |
|  |  | 117 Occupations in forestry, hunting and landscape preservation |
|  | 12 Occupations in gardening and floristry | 121 Occupations in gardening |
|  |  | 122 Occupations in floristry |
| S12 Manufacturing occupations | 21 Occupations in production and processing of raw materials, glass- and ceramic-making and -processing | 211 Occupations in underground and surface mining and blasting engineering |
|  |  | 212 Conditioning and processing of natural stone and minerals, production of building materials |
|  |  | 213 Occupations in industrial glass-making and -processing |
|  |  | 214 Occupations in industrial ceramic-making and -processing |
|  | 22 Occupations in plastic-making and -processing, and wood-working and -processing | 221 Occupations in plastic- and rubber-making and -processing |
|  |  | 222 Occupations in colour coating and varnishing |
|  |  | 223 Occupations in wood-working and -processing |
|  | 23 Occupations in paper-making and -processing, printing, and in technical media design | 231 Technical occupations in paper-making and -processing and packaging |
|  |  | 232 Occupations in technical media design |
|  |  | 233 Occupations in photography and photographic technology |
|  |  | 234 Occupations in printing technology, print finishing, and book binding |
|  | 24 Occupations in metal-making and -working, and in metal construction | 241 Occupations in metal-making |
|  |  | 242 Occupations in metalworking |
|  |  | 243 Occupations in treatment of metal surfaces |
|  |  | 244 Occupations in metal constructing and welding |
|  |  | 245 Occupations in precision mechanics and tool making |
|  | 28 Occupations in textile- and leather-making and -processing | 281 Occupations in textile making |
|  |  | 282 Occupations in the production of clothing and other textile products |
|  |  | 283 Occupations in leather- and fur-making and -processing |
|  | 93 Occupations in product design, artisan craftwork, fine arts and the making of musical instrument | 931 Occupations in product and industrial design |
|  |  | 932 Occupations in interior design, visual marketing, and interior decoration |
|  |  | 933 Occupations in artisan craftwork and fine arts |
|  |  | 934 Artisans designing ceramics and glassware |
|  |  | 935 Artisans working with metal |
|  |  | 936 Occupations in musical instrument making |
| S13 Occupations concerned with production technology | 25 Technical occupations in machine-building and automotive industry | 251 Occupations in machine-building and -operating |
|  |  | 252 Technical occupations in the automotive, aeronautic, aerospace and ship building industries |
|  | 26 Occupations in mechatronics, energy electronics and electrical engineering | 261 Occupations in mechatronics, automation and control technology |
|  |  | 262 Technical occupations in energy technologies |
|  |  | 263 Occupations in electrical engineering |
|  | 27 Occupations in technical research and development, construction, and production planning and scheduling | 271 Occupations in technical research and development |
|  |  | 272 Draftspersons, technical designers, and model makers |
|  |  | 273 Technical occupations in production planning and scheduling |
| S14 Occupations in building and interior construction | 31 Occupations in construction scheduling, architecture and surveying | 311 Occupations in construction scheduling and supervision, and architecture |
|  |  | 312 Occupations in surveying and cartography |
|  | 32 Occupations in building construction above and below ground | 321 Occupations in building construction |
|  |  | 322 Occupations in civil engineering |
|  | 33 Occupations in interior construction | 331 Floor layers |
|  |  | 332 Painters and varnishers, plasterers, occupations in the waterproofing of buildings, preservation of structures and wooden building components |
|  |  | 333 Occupations in the interior construction and dry walling, insulation, carpentry, glazing, roller shutter and jalousie installation |
|  | 34 Occupations in building services engineering and technical building services | 341 Occupations in building services engineering |
|  |  | 342 Occupations in plumping, sanitation, heating, ventilating, and air conditioning |
|  |  | 343 Occupations in building services and waste disposal |
| S21 Occupations in the food industry, in gastronomy and in tourism | 29 Occupations in food-production and -processing | 291 Occupations in beverage production |
|  |  | 292 Occupations in the production of foodstuffs, confectionery and tobacco products |
|  |  | 293 Cooking occupations |
|  | 63 Occupations in tourism, hotels and restaurants | 631 Occupations in tourism and the sports (and fitness) industry |
|  |  | 632 Occupations in hotels |
|  |  | 633 Gastronomy occupations |
|  |  | 634 Occupations in event organisation and management |
| S22 Medical and non-medical health care occupations | 81 Medical and health care occupations | 811 Doctors’ receptionists and assistants |
|  |  | 812 Laboratory occupations in medicine |
|  |  | 813 Occupations in nursing, emergency medical services and obstetrics |
|  |  | 814 Occupations in human medicine and dentistry |
|  |  | 815 Occupations in veterinary medicine and non-medical animal health practitioners |
|  |  | 816 Occupations in psychology and non-medical psychotherapy |
|  |  | 817 Occupations in non-medical therapy and alternative medicine |
|  |  | 818 Occupations in pharmacy |
|  | 82 Occupations in non-medical healthcare, body care, wellness and medical technicians | 821 Occupations in geriatric care |
|  |  | 822 Occupations providing nutritional advice or health counselling, and occupations in wellness |
|  |  | 823 Occupations in body care |
|  |  | 824 Occupations in funeral services |
|  |  | 825 Technical occupations in medicine, orthopaedic and rehabilitation |
| S23 Service occupations in social sector and cultural work | 83 Occupations in education and social work, housekeeping, and theology | 831 Occupations in education and social work, and pedagogic specialists in social care work |
|  |  | 832 Occupations in housekeeping and consumer counselling |
|  |  | 833 Occupations in theology and church community work |
|  | 84 Occupations in teaching and training | 841 Teachers in schools of general education |
|  |  | 842 Teachers for occupation-specific subjects at vocational schools and in-company instructors in vocational training |
|  |  | 843 Teachers and researcher at universities and colleges |
|  |  | 844 Teachers at educational institutions other than schools (except driving, flying and sports instructors) |
|  |  | 845 Driving, flying and sports instructors at educational institutions other than schools |
|  | 91 Occupations in in philology, literature, humanities, social sciences, and economics | 911 Occupations in philology |
|  |  | 912 Occupations in the humanities |
|  |  | 913 Occupations in the social sciences |
|  |  | 914 Occupations in economics |
|  | 94 Occupations in the performing arts and entertainment | 941 Musicians, singers and conductors |
|  |  | 942 Actors, dancers, athletes and related occupations |
|  |  | 943 Presenters and entertainers |
|  |  | 944 Occupations in theatre, film and television productions |
|  |  | 945 Occupations in event technology, cinematography, and sound engineering |
|  |  | 946 Occupations in stage, costume and prop design |
|  |  | 947 Technical and management occupations in museums and exhibitions |
| S31 Occupations in commerce and trade | 61 Occupations in purchasing, sales and trading | 611 Occupations in purchasing and sales |
|  |  | 612 Trading occupations |
|  |  | 613 Occupations in real estate and facility management |
|  | 62 Sales occupations in retail trade | 621 Sales occupations in retail trade (without product specialisation) |
|  |  | 622 Sales occupations (retail trade) selling clothing, electronic devices, furniture, motor vehicles and other durables |
|  |  | 623 Sales occupations (retail) selling foodstuffs |
|  |  | 624 Sales occupations (retail) selling drugstore products, pharmaceuticals, medical supplies and healthcare goods |
|  |  | 625 Sales occupations (retail) selling books, art, antiques, musical instruments, recordings or sheet music |
| S32 Occupations in business management and organisation | 71 Occupations in business management and organisation | 711 Managing directors and executive board members |
|  |  | 712 Legislators and senior officials of special interest organisations |
|  |  | 713 Occupations in business organisation and strategy |
|  |  | 714 Office clerks and secretaries |
|  |  | 715 Occupations in human resources management and personnel service |
| S33 Business related service occupations | 72 Occupations in financial services, accounting and tax consultancy | 721 Occupations in insurance and financial services |
|  |  | 722 Occupations in accounting, controlling and auditing |
|  |  | 723 Occupations in tax consultancy |
|  | 73 Occupations in law and public administration | 731 Occupations in legal services, jurisdiction, and other officers of the court |
|  |  | 732 Occupations in public administration |
|  |  | 733 Occupations in media, documentation and information services |
|  | 92 Occupations in advertising and marketing, in commercial and editorial media design | 921 Occupations in advertising and marketing |
|  |  | 922 Occupations in public relations |
|  |  | 923 Occupations in publishing and media management |
|  |  | 924 Occupations in editorial work and journalism |
| S41 Service occupations in the IT-sector and the natural sciences | 41 Occupations in mathematics, biology, chemistry and physics | 411 Occupations in mathematics and statistics |
|  |  | 412 Occupations in biology |
|  |  | 413 Occupations in chemistry |
|  |  | 414 Occupations in physics |
|  | 42 Occupations in geology, geography and environmental protection | 421 Occupations in geology, geography and meteorology |
|  |  | 422 Occupations in environmental protection engineering |
|  |  | 423 Occupations in environmental protection management and environmental protection consulting |
|  | 43 Occupations in computer science, information and communication technology | 431 Occupations in computer science |
|  |  | 432 Occupations in IT-system-analysis, IT-application-consulting and IT-sales |
|  |  | 433 Occupations in IT-network engineering, IT-coordination, IT-administration and IT-organisation |
|  |  | 434 Occupations in software development and programming |
| S51 Safety and security occupations | 53 Occupations in safety and health protection, security and surveillance | 531 Occupations in physical security, personal protection, fire protection and workplace safety |
|  |  | 532 Occupations in police and criminal investigation, jurisdiction and the penal institution |
|  |  | 533 Occupations in occupational health and safety administration, public health authority, and disinfection |
|  | 01 Armed forces personnel | 011 Commissioned officers |
|  |  | 012 Senior non-commissioned officers and higher |
|  |  | 013 Junior non-commissioned officers |
|  |  | 014 Armed forces personnel in other ranks |
| S52 Occupations in traffic and logistics | 51 Occupations in traffic and logistics (without vehicle driving) | 511 Technical occupations in railway, aircraft and ship operation |
|  |  | 512 Occupations in the inspection and maintenance of traffic infrastructure |
|  |  | 513 Occupations in warehousing and logistics, in postal and other delivery services, and in cargo handling |
|  |  | 514 Service occupations in passenger traffic |
|  |  | 515 Occupations in traffic surveillance and control |
|  |  | 516 Management assistants in transport and logistics |
|  | 52 Drivers and operators of vehicles and transport equipment | 521 Driver of vehicles in road traffic |
|  |  | 522 Drivers of vehicles in railway traffic |
|  |  | 523 Aircraft pilots |
|  |  | 524 Ship’s officers and masters |
|  |  | 525 Drivers and operators of construction and transportation vehicles and equipment |
| S53 Occupations in cleaning services | 54 Occupations in cleaning services | 541 Occupations in cleaning services |

Source: Matthes et al., 2015
