# Supplementary material for: Occupational differences in sickness presenteeism in the baseline survey of the German National Cohort (NAKO)
Source: J Occup Med Toxicol. 2026 Sep 25;21:31. doi: 10.1186/s12995-026-00529-1 (PMC13615606; doi:10.1186/s12995-026-00529-1)
Supplement: Supplementary file 2 [file 12995_2026_529_MOESM2_ESM.docx]

**Additional file 2:** Prevalence ratios (PRs) and 95% confidence intervals (CIs) for sickness presenteeism (SP) by occupational segments (full results)

| **Occupational segment** | **SP**  **N (%)** | **Model 1**  **PR (95% CI)** | **Model 2**  **(main model)**  **PR (95% CI)** | **Model 3**  **PR (95% CI)** | **Model 4**  **PR (95% CI)** |
| --- | --- | --- | --- | --- | --- |
|  |  | (adjusted for age, sex) | (adjusted for Model 1, educational level, health status) | (adjusted for Model 2, migration background, income, marital status, the presence of children) | (adjusted for Model 2, paid working time, night shift work) |
| Medical and non-medical health care occupations | 10,195 (82.6%) | 1.07 (1.06–1.08) | 1.07 (1.06–1.08) | 1.07 (1.06–1.08) | 1.06 (1.05–1.07) |
| Occupations in the food industry, in gastronomy and in tourism | 2,018 (82.3%) | 1.07 (1.05–1.09) | 1.07 (1.04–1.09) | 1.07 (1.05–1.09) | 1.06 (1.04–1.08) |
| Occupations in commerce and trade | 6,274 (80.8%) | 1.06 (1.05–1.07) | 1.06 (1.04–1.07) | 1.06 (1.05–1.07) | 1.06 (1.05–1.08) |
| Service occupations in social sector and cultural work | 12,861 (80.3%) | 1.05 (1.04–1.06) | 1.05 (1.04–1.06) | 1.05 (1.04–1.06) | 1.06 (1.05–1.07) |
| Occupations in business management and organisation | 13,315 (75.8%) | 0.98 (0.97–0.99) | 0.98 (0.97–0.99) | 0.98 (0.97–0.99) | 0.99 (0.98–1.00) |
| Business related service occupations | 12,990 (75.2%) | 0.97 (0.96–0.98) | 0.97 (0.96–0.98) | 0.97 (0.96–0.98) | 0.98 (0.97–0.99) |
| Occupations in traffic and logistics | 4,090 (74.8%) | 1.00 (0.98–1.02) | 0.99 (0.98–1.01) | 1.00 (0.98–1.01) | 0.98 (0.96–0.99) |
| Safety and security occupations | 2,271 (74.2%) | 0.99 (0.97–1.02) | 1.00 (0.97–1.02) | 0.99 (0.97–1.02) | 0.95 (0.92–0.97) |
| Occupations in building and interior construction | 3,527 (73.8%) | 0.99 (0.97–1.01) | 0.99 (0.97–1.01) | 0.99 (0.97–1.01) | 1.00 (0.98–1.02) |
| Manufacturing occupations | 2,315 (72.1%) | 0.96 (0.94–0.99) | 0.96 (0.93–0.98) | 0.96 (0.93–0.98) | 0.96 (0.93–0.98) |
| Occupations concerned with production technology | 8,477 (72.0%) | 0.96 (0.95–0.98) | 0.96 (0.95–0.98) | 0.96 (0.95–0.98) | 0.96 (0.95–0.98) |
| Occupations in cleaning services | 500 (71.2%) | 0.92 (0.87–0.97) | 0.91 (0.86–0.97) | 0.92 (0.87–0.97) | 0.93 (0.88–0.98) |
| Occupations in agriculture, forestry and horticulture | 707 (71.0%) | 0.94 (0.89–0.98) | 0.93 (0.89–0.98) | 0.94 (0.89–0.98) | 0.94 (0.90–0.98) |
| Service occupations in the IT-sector and the natural sciences | 5,190 (67.6%) | 0.89 (0.87–0.91) | 0.90 (0.88–0.91) | 0.90 (0.88–0.91) | 0.90 (0.89–0.92) |

Model estimates are presented as prevalence ratios (95% confidence intervals) and are arranged in descending order based on Model 2 results. Missing values (n = 2,125) are not presented in the table.
