# Supplementary material for: Occupational differences in sickness presenteeism in the baseline survey of the German National Cohort (NAKO)
Source: J Occup Med Toxicol. 2026 Sep 25;21:31. doi: 10.1186/s12995-026-00529-1 (PMC13615606; doi:10.1186/s12995-026-00529-1)
Supplement: Supplementary file 3 [file 12995_2026_529_MOESM3_ESM.docx]

**Additional file 3:** Prevalence ratios (PRs) and 95% confidence intervals (CIs) for sickness presenteeism (SP) by occupational groups

| **Code** | **Occupational group** | **N** | **SP prevalence (%)** | **Model 1**  **PR (95% CI)** | **Model 2**  **(main model)**  **PR (95% CI)** | **Model 3**  **PR (95% CI)** | **Model 4**  **PR (95% CI)** |
| --- | --- | --- | --- | --- | --- | --- | --- |
|  |  |  |  | (adjusted for age, sex) | (adjusted for Model 1, educational level, health status) | (adjusted for Model 2, migration background, income, marital status, the presence of children) | (adjusted for Model 2, paid working time, night shift work) |
| 946 | Occupations in stage, costume and prop design, | 31 | 93.5% | 1.21 (1.10–1.33) | 1.24 (1.14–1.36) | 1.25 (1.14–1.36) | 1.23 (1.13–1.35) |
| 113 | Occupations in horsekeeping | 10 | 100.0% | 1.28 (1.25–1.30) | 1.23 (1.18–1.29) | 1.24 (1.18–1.30) | 1.27 (1.21–1.33) |
| 291 | Occupations in beverage production | 36 | 91.7% | 1.24 (1.13–1.37) | 1.23 (1.11–1.37) | 1.23 (1.11–1.36) | 1.22 (1.10–1.36) |
| 815 | Occupations in veterinary medicine and non-medical animal health practitioners | 75 | 94.7% | 1.20 (1.14–1.27) | 1.20 (1.14–1.27) | 1.20 (1.14–1.27) | 1.19 (1.12–1.26) |
| 814 | Occupations in human medicine and dentistry | 1,340 | 87.7% | 1.14 (1.12–1.17) | 1.17 (1.15–1.20) | 1.17 (1.14–1.19) | 1.12 (1.09–1.14) |
| 115 | Occupations in animal care | 35 | 91.4% | 1.19 (1.07–1.32) | 1.17 (1.05–1.30) | 1.17 (1.06–1.31) | 1.17 (1.05–1.30) |
| 942 | Actors, dancers, athletes and related occupations | 32 | 84.4% | 1.13 (0.97–1.31) | 1.17 (1.00–1.36) | 1.16 (1.00–1.35) | 1.20 (1.03–1.39) |
| 941 | Musicians, singers and conductors | 186 | 84.9% | 1.12 (1.06–1.19) | 1.14 (1.07–1.21) | 1.14 (1.07–1.21) | 1.15 (1.09–1.23) |
| 212 | Conditioning and processing of natural stone and minerals, production of building materials | 17 | 82.4% | 1.12 (0.90–1.40) | 1.14 (0.91–1.42) | 1.14 (0.91–1.42) | 1.11 (0.88–1.40) |
| 711 | Managing directors and executive board members | 887 | 82.5% | 1.11 (1.07–1.14) | 1.12 (1.08–1.15) | 1.11 (1.08–1.15) | 1.09 (1.06–1.12) |
| 841 | Teachers in schools of general education | 4,015 | 83.7% | 1.09 (1.08–1.11) | 1.10 (1.09–1.12) | 1.10 (1.08–1.12) | 1.11 (1.09–1.12) |
| 283 | Occupations in leather- and fur-making and -processing | 36 | 83.3% | 1.11 (0.96–1.28) | 1.10 (0.95–1.27) | 1.10 (0.95–1.27) | 1.10 (0.96–1.27) |
| 813 | Occupations in nursing, emergency medical services and obstetrics | 3,337 | 83.8% | 1.08 (1.06–1.09) | 1.08 (1.06–1.09) | 1.08 (1.06–1.09) | 1.04 (1.03–1.06) |
| 621 | Sales occupations in retail trade (without product specialisation) | 2,349 | 84.3% | 1.09 (1.07–1.11) | 1.08 (1.06–1.10) | 1.08 (1.07–1.10) | 1.10 (1.08–1.12) |
| 633 | Gastronomy occupations | 549 | 83.8% | 1.08 (1.04–1.12) | 1.08 (1.04–1.12) | 1.08 (1.04–1.12) | 1.06 (1.02–1.10) |
| 622 | Sales occupations (retail trade) selling clothing, electronic devices, furniture, motor vehicles and other durables | 397 | 81.6% | 1.08 (1.03–1.13) | 1.08 (1.03–1.13) | 1.08 (1.03–1.13) | 1.08 (1.03–1.13) |
| 516 | Management assistants in transport and logistics | 759 | 80.6% | 1.07 (1.03–1.11) | 1.07 (1.03–1.10) | 1.07 (1.03–1.10) | 1.06 (1.03–1.10) |
| 842 | Teachers for occupation-specific subjects at vocational schools and in-company instructors in vocational training | 651 | 81.6% | 1.07 (1.03–1.11) | 1.07 (1.03–1.11) | 1.07 (1.03–1.11) | 1.08 (1.04–1.12) |
| 293 | Cooking occupations | 537 | 82.9% | 1.09 (1.05–1.13) | 1.07 (1.03–1.11) | 1.08 (1.04–1.12) | 1.08 (1.04–1.12) |
| 823 | Occupations in body care | 261 | 86.2% | 1.09 (1.04–1.14) | 1.07 (1.02–1.13) | 1.08 (1.03–1.14) | 1.10 (1.04–1.15) |
| 811 | Doctors’ receptionists and assistants | 1,296 | 83.9% | 1.06 (1.04–1.09) | 1.06 (1.03–1.09) | 1.06 (1.04–1.09) | 1.08 (1.05–1.10) |
| 821 | Occupations in geriatric care | 761 | 83.6% | 1.07 (1.04–1.11) | 1.06 (1.02–1.09) | 1.06 (1.03–1.09) | 1.04 (1.01–1.08) |
| 292 | Occupations in the production of foodstuffs, confectionery and tobacco products | 256 | 80.9% | 1.07 (1.01–1.14) | 1.06 (1.00–1.13) | 1.06 (1.00–1.13) | 1.03 (0.97–1.09) |
| 234 | Occupations in printing technology, print finishing, and book binding | 147 | 79.6% | 1.07 (0.98–1.16) | 1.06 (0.98–1.15) | 1.06 (0.98–1.15) | 1.04 (0.96–1.13) |
| 624 | Sales occupations (retail) selling drugstore products, pharmaceuticals, medical supplies and healthcare goods | 101 | 84.2% | 1.07 (0.98–1.16) | 1.06 (0.97–1.15) | 1.06 (0.97–1.15) | 1.07 (0.98–1.16) |
| 831 | Occupations in education and social work, and pedagogic specialists in social care work | 4,418 | 82.5% | 1.06 (1.05–1.08) | 1.05 (1.04–1.07) | 1.05 (1.04–1.07) | 1.06 (1.04–1.07) |
| 623 | Sales occupations (retail) selling foodstuffs | 304 | 83.2% | 1.06 (1.01–1.11) | 1.05 (1.00–1.10) | 1.05 (1.00–1.11) | 1.05 (1.00–1.10) |
| 632 | Occupations in hotels | 215 | 80.5% | 1.03 (0.97–1.10) | 1.05 (0.98–1.12) | 1.05 (0.98–1.12) | 1.03 (0.97–1.10) |
| 634 | Occupations in event organisation and management | 172 | 82.0% | 1.05 (0.98–1.12) | 1.05 (0.98–1.13) | 1.05 (0.98–1.13) | 1.03 (0.96–1.11) |
| 241 | Occupations in metal-making | 68 | 77.9% | 1.05 (0.93–1.19) | 1.05 (0.93–1.19) | 1.05 (0.93–1.19) | 1.02 (0.90–1.15) |
| 611 | Occupations in purchasing and sales | 2,074 | 77.7% | 1.03 (1.01–1.06) | 1.04 (1.02–1.07) | 1.04 (1.02–1.06) | 1.04 (1.01–1.06) |
| 521 | Driver of vehicles in road traffic | 1,123 | 76.9% | 1.05 (1.02–1.08) | 1.04 (1.01–1.07) | 1.04 (1.01–1.08) | 0.99 (0.96–1.03) |
| 252 | Technical occupations in the automotive, aeronautic, aerospace and ship building industries | 850 | 77.1% | 1.05 (1.01–1.08) | 1.04 (1.00–1.07) | 1.04 (1.00–1.07) | 1.04 (1.00–1.08) |
| 825 | Technical occupations in medicine, orthopaedic and rehabilitation | 413 | 79.4% | 1.04 (0.99–1.09) | 1.04 (0.99–1.09) | 1.04 (0.99–1.10) | 1.05 (1.00–1.11) |
| 945 | Occupations in event technology, cinematography, and sound engineering | 153 | 78.4% | 1.05 (0.96–1.14) | 1.04 (0.96–1.13) | 1.05 (0.96–1.14) | 1.03 (0.95–1.12) |
| 531 | Occupations in physical security, personal protection, fire protection and workplace safety | 732 | 76.5% | 1.03 (0.99–1.08) | 1.03 (0.99–1.07) | 1.03 (0.99–1.07) | 0.97 (0.93–1.01) |
| 612 | Trading occupations | 449 | 78.0% | 1.02 (0.98–1.08) | 1.03 (0.98–1.08) | 1.03 (0.98–1.08) | 1.03 (0.98–1.08) |
| 631 | Occupations in tourism and the sports (and fitness) industry | 253 | 79.8% | 1.03 (0.96–1.09) | 1.03 (0.97–1.10) | 1.03 (0.97–1.09) | 1.04 (0.98–1.11) |
| 712 | Legislators and senior officials of special interest organisations | 193 | 76.7% | 1.02 (0.94–1.10) | 1.03 (0.95–1.11) | 1.03 (0.95–1.11) | 1.02 (0.95–1.10) |
| 112 | Occupations in animal husbandry | 33 | 78.8% | 1.04 (0.87–1.24) | 1.03 (0.87–1.23) | 1.04 (0.87–1.23) | 1.01 (0.85–1.20) |
| 817 | Occupations in non-medical therapy and alternative medicine | 997 | 79.9% | 1.02 (0.99–1.05) | 1.02 (0.99–1.06) | 1.03 (0.99–1.06) | 1.04 (1.01–1.08) |
| 342 | Occupations in plumping, sanitation, heating, ventilating, and air conditioning | 336 | 75.3% | 1.02 (0.96–1.09) | 1.02 (0.96–1.08) | 1.02 (0.96–1.08) | 1.03 (0.97–1.09) |
| 332 | Painters and varnishers, plasterers, occupations in the waterproofing of buildings, preservation of structures and wooden building components | 161 | 77.0% | 1.04 (0.96–1.13) | 1.02 (0.94–1.11) | 1.03 (0.94–1.11) | 1.04 (0.95–1.13) |
| 282 | Occupations in the production of clothing and other textile products | 88 | 80.7% | 1.03 (0.93–1.14) | 1.02 (0.92–1.12) | 1.02 (0.92–1.13) | 1.04 (0.94–1.15) |
| 331 | Floor layers | 60 | 75.0% | 1.02 (0.88–1.18) | 1.02 (0.88–1.18) | 1.02 (0.88–1.18) | 1.03 (0.89–1.19) |
| 715 | Occupations in human resources management and personnel service | 1,074 | 78.6% | 1.01 (0.98–1.04) | 1.01 (0.98–1.04) | 1.01 (0.98–1.04) | 1.01 (0.98–1.05) |
| 613 | Occupations in real estate and facility management | 506 | 77.3% | 1.01 (0.96–1.06) | 1.01 (0.96–1.06) | 1.01 (0.96–1.06) | 1.02 (0.97–1.07) |
| 923 | Occupations in publishing and media management | 109 | 78.0% | 1.01 (0.92–1.11) | 1.01 (0.91–1.11) | 1.01 (0.91–1.11) | 1.01 (0.92–1.12) |
| 944 | Occupations in theatre, film and television productions | 97 | 77.3% | 1.01 (0.90–1.12) | 1.01 (0.91–1.12) | 1.01 (0.91–1.12) | 0.97 (0.87–1.07) |
| 932 | Occupations in interior design, visual marketing, and interior decoration | 88 | 78.4% | 1.01 (0.91–1.13) | 1.01 (0.91–1.13) | 1.02 (0.91–1.14) | 1.02 (0.91–1.14) |
| 222 | Occupations in colour coating and varnishing | 79 | 75.9% | 1.02 (0.91–1.16) | 1.01 (0.89–1.14) | 1.01 (0.89–1.14) | 1.01 (0.90–1.14) |
| 731 | Occupations in legal services, jurisdiction, and other officers of the court | 1,040 | 76.9% | 1.00 (0.96–1.03) | 1.00 (0.97–1.03) | 1.00 (0.97–1.03) | 1.00 (0.97–1.04) |
| 311 | Occupations in construction scheduling and supervision, and architecture | 1,359 | 75.1% | 1.00 (0.97–1.03) | 1.00 (0.97–1.03) | 1.00 (0.97–1.03) | 1.01 (0.98–1.04) |
| 844 | Teachers at educational institutions other than schools (except driving, flying and sports instructors) | 457 | 75.9% | 0.99 (0.94–1.05) | 1.00 (0.95–1.05) | 1.00 (0.95–1.06) | 1.00 (0.95–1.06) |
| 321 | Occupations in building construction | 312 | 74.4% | 1.01 (0.94–1.08) | 1.00 (0.94–1.07) | 1.00 (0.94–1.07) | 1.00 (0.94–1.07) |
| 833 | Occupations in theology and church community work | 209 | 74.2% | 0.99 (0.92–1.08) | 1.00 (0.92–1.08) | 1.00 (0.92–1.08) | 0.97 (0.90–1.05) |
| 845 | Driving, flying and sports instructors at educational institutions other than schools | 165 | 75.2% | 0.99 (0.91–1.08) | 1.00 (0.91–1.09) | 1.00 (0.92–1.09) | 1.00 (0.92–1.09) |
| 111 | Occupations in farming | 138 | 75.4% | 1.00 (0.91–1.10) | 1.00 (0.91–1.10) | 1.00 (0.91–1.10) | 0.98 (0.89–1.08) |
| 822 | Occupations providing nutritional advice or health counselling, and occupations in wellness | 102 | 78.4% | 1.00 (0.91–1.11) | 1.00 (0.91–1.11) | 1.00 (0.91–1.11) | 1.01 (0.92–1.12) |
| 713 | Occupations in business organisation and strategy | 6,218 | 75.5% | 0.99 (0.97–1.00) | 0.99 (0.97–1.00) | 0.99 (0.97–1.00) | 0.99 (0.98–1.01) |
| 722 | Occupations in accounting, controlling and auditing | 2,020 | 76.5% | 0.99 (0.96–1.01) | 0.99 (0.97–1.01) | 0.99 (0.96–1.01) | 1.00 (0.97–1.02) |
| 532 | Occupations in police and criminal investigation, jurisdiction and the penal institution | 1,197 | 73.9% | 0.98 (0.95–1.02) | 0.99 (0.96–1.02) | 0.99 (0.96–1.02) | 0.94 (0.91–0.98) |
| 812 | Laboratory occupations in medicine | 545 | 77.6% | 0.99 (0.95–1.04) | 0.99 (0.94–1.03) | 0.99 (0.95–1.03) | 0.98 (0.93–1.02) |
| 262 | Technical occupations in energy technologies | 579 | 73.2% | 1.00 (0.95–1.05) | 0.99 (0.94–1.04) | 0.99 (0.95–1.04) | 0.99 (0.94–1.04) |
| 261 | Occupations in mechatronics, automation and control technology | 180 | 73.3% | 0.99 (0.91–1.08) | 0.99 (0.91–1.08) | 0.99 (0.91–1.08) | 0.99 (0.91–1.08) |
| 221 | Occupations in plastic- and rubber-making and -processing | 62 | 74.2% | 1.00 (0.86–1.16) | 0.99 (0.86–1.15) | 1.00 (0.86–1.16) | 0.98 (0.84–1.13) |
| 251 | Occupations in machine-building and -operating | 1,953 | 73.4% | 0.99 (0.96–1.02) | 0.98 (0.96–1.01) | 0.99 (0.96–1.01) | 0.98 (0.95–1.00) |
| 921 | Occupations in advertising and marketing | 1,495 | 75.6% | 0.98 (0.95–1.01) | 0.98 (0.95–1.01) | 0.98 (0.95–1.01) | 0.98 (0.95–1.01) |
| 433 | Occupations in IT-network engineering, IT-coordination, IT-administration and IT-organisation | 756 | 72.9% | 0.98 (0.94–1.02) | 0.98 (0.94–1.02) | 0.98 (0.94–1.02) | 0.99 (0.95–1.03) |
| 818 | Occupations in pharmacy | 674 | 75.8% | 0.97 (0.93–1.01) | 0.98 (0.94–1.02) | 0.98 (0.94–1.02) | 0.98 (0.94–1.02) |
| 924 | Occupations in editorial work and journalism | 549 | 75.0% | 0.98 (0.93–1.03) | 0.98 (0.94–1.03) | 0.99 (0.94–1.03) | 0.98 (0.94–1.03) |
| 341 | Occupations in building services engineering | 584 | 72.4% | 0.99 (0.94–1.04) | 0.98 (0.93–1.03) | 0.98 (0.93–1.03) | 1.00 (0.95–1.05) |
| 244 | Occupations in metal constructing and welding | 375 | 72.5% | 0.99 (0.93–1.05) | 0.98 (0.92–1.04) | 0.98 (0.92–1.04) | 0.98 (0.92–1.04) |
| 343 | Occupations in building services and waste disposal | 247 | 73.3% | 0.99 (0.92–1.07) | 0.98 (0.91–1.06) | 0.98 (0.91–1.06) | 0.98 (0.91–1.05) |
| 322 | Occupations in civil engineering | 157 | 73.2% | 0.99 (0.90–1.09) | 0.98 (0.89–1.08) | 0.98 (0.89–1.08) | 0.97 (0.88–1.06) |
| 625 | Sales occupations (retail) selling books, art, antiques, musical instruments, recordings or sheet music | 94 | 76.6% | 0.99 (0.88–1.11) | 0.98 (0.87–1.10) | 0.98 (0.88–1.11) | 0.99 (0.88–1.12) |
| 011 | Commissioned officers | 19 | 73.7% | 0.98 (0.75–1.28) | 0.98 (0.75–1.28) | 0.98 (0.75–1.28) | 0.94 (0.71–1.24) |
| 721 | Occupations in insurance and financial services | 2,693 | 74.4% | 0.97 (0.95–0.99) | 0.97 (0.95–0.99) | 0.97 (0.95–0.99) | 0.98 (0.96–1.01) |
| 513 | Occupations in warehousing and logistics, in postal and other delivery services, and in cargo handling | 1,406 | 74.1% | 0.98 (0.95–1.01) | 0.97 (0.94–1.00) | 0.98 (0.95–1.01) | 0.98 (0.95–1.01) |
| 723 | Occupations in tax consultancy | 588 | 76.4% | 0.98 (0.93–1.02) | 0.97 (0.93–1.02) | 0.97 (0.93–1.02) | 0.98 (0.94–1.03) |
| 922 | Occupations in public relations | 239 | 74.1% | 0.95 (0.89–1.03) | 0.97 (0.90–1.05) | 0.97 (0.90–1.05) | 0.97 (0.91–1.05) |
| 122 | Occupations in floristry | 76 | 76.3% | 0.97 (0.86–1.10) | 0.97 (0.85–1.09) | 0.97 (0.86–1.10) | 0.99 (0.87–1.12) |
| 732 | Occupations in public administration | 3,880 | 74.5% | 0.96 (0.95–0.98) | 0.96 (0.94–0.98) | 0.96 (0.94–0.98) | 0.96 (0.95–0.98) |
| 273 | Technical occupations in production planning and scheduling | 2,276 | 71.6% | 0.96 (0.93–0.98) | 0.96 (0.94–0.99) | 0.96 (0.93–0.98) | 0.97 (0.94–0.99) |
| 843 | Teachers and researcher at universities and colleges | 1,550 | 71.9% | 0.94 (0.91–0.97) | 0.96 (0.93–0.99) | 0.96 (0.93–0.99) | 0.95 (0.92–0.98) |
| 413 | Occupations in chemistry | 743 | 72.8% | 0.96 (0.92–1.00) | 0.96 (0.92–1.00) | 0.96 (0.92–1.00) | 0.96 (0.92–1.00) |
| 832 | Occupations in housekeeping and consumer counselling | 317 | 76.0% | 0.97 (0.91–1.03) | 0.96 (0.90–1.02) | 0.97 (0.91–1.03) | 0.99 (0.93–1.05) |
| 414 | Occupations in physics | 238 | 70.2% | 0.95 (0.87–1.03) | 0.96 (0.88–1.04) | 0.95 (0.88–1.04) | 0.96 (0.89–1.05) |
| 943 | Presenters and entertainers | 24 | 70.8% | 0.94 (0.73–1.23) | 0.96 (0.74–1.26) | 0.96 (0.73–1.26) | 0.94 (0.72–1.22) |
| 816 | Occupations in psychology and non-medical psychotherapy | 357 | 73.7% | 0.94 (0.89–1.00) | 0.95 (0.89–1.01) | 0.95 (0.89–1.01) | 0.96 (0.90–1.02) |
| 522 | Drivers of vehicles in railway traffic | 126 | 69.8% | 0.95 (0.85–1.06) | 0.95 (0.85–1.06) | 0.95 (0.85–1.06) | 0.89 (0.79–1.00) |
| 533 | Occupations in occupational health and safety administration, public health authority, and disinfection | 59 | 72.9% | 0.95 (0.81–1.11) | 0.95 (0.82–1.11) | 0.95 (0.82–1.11) | 0.96 (0.82–1.11) |
| 714 | Office clerks and secretaries | 4,943 | 74.4% | 0.95 (0.93–0.96) | 0.94 (0.93–0.96) | 0.94 (0.93–0.96) | 0.96 (0.94–0.97) |
| 232 | Occupations in technical media design | 331 | 72.8% | 0.94 (0.88–1.01) | 0.94 (0.88–1.01) | 0.94 (0.88–1.01) | 0.95 (0.89–1.01) |
| 412 | Occupations in biology | 331 | 72.2% | 0.93 (0.87–1.00) | 0.94 (0.88–1.01) | 0.94 (0.88–1.01) | 0.95 (0.89–1.02) |
| 512 | Occupations in the inspection and maintenance of traffic infrastructure | 82 | 69.5% | 0.94 (0.81–1.09) | 0.94 (0.81–1.09) | 0.94 (0.81–1.09) | 0.92 (0.80–1.07) |
| 933 | Occupations in artisan craftwork and fine arts | 39 | 71.8% | 0.93 (0.77–1.14) | 0.94 (0.77–1.14) | 0.94 (0.77–1.15) | 0.95 (0.78–1.16) |
| 263 | Occupations in electrical engineering | 1,200 | 69.0% | 0.93 (0.90–0.97) | 0.93 (0.90–0.97) | 0.93 (0.90–0.97) | 0.94 (0.91–0.98) |
| 271 | Occupations in technical research and development | 936 | 69.0% | 0.92 (0.88–0.96) | 0.93 (0.89–0.97) | 0.93 (0.89–0.97) | 0.94 (0.90–0.98) |
| 733 | Occupations in media, documentation and information services | 377 | 72.7% | 0.94 (0.88–1.00) | 0.93 (0.88–0.99) | 0.93 (0.88–0.99) | 0.94 (0.88–1.00) |
| 242 | Occupations in metalworking | 331 | 69.5% | 0.94 (0.87–1.01) | 0.93 (0.87–1.00) | 0.93 (0.87–1.00) | 0.92 (0.86–0.99) |
| 014 | Armed forces personnel in other ranks | 262 | 69.5% | 0.93 (0.86–1.01) | 0.93 (0.86–1.01) | 0.93 (0.86–1.01) | 0.90 (0.83–0.97) |
| 231 | Technical occupations in paper-making and -processing and packaging | 34 | 67.6% | 0.92 (0.73–1.16) | 0.93 (0.74–1.17) | 0.93 (0.74–1.18) | 0.91 (0.72–1.15) |
| 935 | Artisans working with metal | 22 | 72.7% | 0.94 (0.73–1.22) | 0.93 (0.72–1.20) | 0.94 (0.73–1.21) | 0.93 (0.73–1.20) |
| 431 | Occupations in computer science | 981 | 68.5% | 0.91 (0.88–0.95) | 0.92 (0.88–0.96) | 0.92 (0.88–0.96) | 0.93 (0.89–0.97) |
| 272 | Draftspersons, technical designers, and model makers | 503 | 70.2% | 0.93 (0.87–0.98) | 0.92 (0.87–0.98) | 0.92 (0.87–0.98) | 0.94 (0.88–0.99) |
| 913 | Occupations in the social sciences | 348 | 71.3% | 0.92 (0.86–0.98) | 0.92 (0.86–0.99) | 0.92 (0.86–0.99) | 0.93 (0.87–0.99) |
| 515 | Occupations in traffic surveillance and control | 204 | 69.1% | 0.93 (0.85–1.02) | 0.92 (0.84–1.01) | 0.92 (0.84–1.01) | 0.89 (0.82–0.98) |
| 824 | Occupations in funeral services | 37 | 70.3% | 0.94 (0.76–1.15) | 0.92 (0.74–1.13) | 0.92 (0.75–1.14) | 0.90 (0.73–1.10) |
| 541 | Occupations in cleaning services | 500 | 71.2% | 0.92 (0.87–0.97) | 0.91 (0.86–0.97) | 0.92 (0.87–0.97) | 0.93 (0.88–0.98) |
| 912 | Occupations in the humanities | 69 | 69.6% | 0.91 (0.78–1.06) | 0.91 (0.77–1.06) | 0.91 (0.78–1.06) | 0.91 (0.78–1.07) |
| 233 | Occupations in photography and photographic technology | 36 | 69.4% | 0.91 (0.73–1.13) | 0.91 (0.73–1.14) | 0.92 (0.74–1.14) | 0.92 (0.75–1.14) |
| 223 | Occupations in wood-working and -processing | 288 | 67.0% | 0.91 (0.84–0.98) | 0.90 (0.83–0.98) | 0.91 (0.84–0.98) | 0.91 (0.84–0.99) |
| 525 | Drivers and operators of construction and transportation vehicles and equipment | 152 | 67.1% | 0.91 (0.82–1.02) | 0.90 (0.81–1.00) | 0.90 (0.81–1.01) | 0.89 (0.80–0.99) |
| 333 | Occupations in the interior construction and dry walling, insulation, carpentry, glazing, roller shutter and jalousie installation | 150 | 66.7% | 0.91 (0.81–1.02) | 0.90 (0.80–1.01) | 0.91 (0.81–1.01) | 0.91 (0.81–1.02) |
| 281 | Occupations in textile making | 22 | 68.2% | 0.90 (0.68–1.20) | 0.90 (0.68–1.20) | 0.91 (0.68–1.21) | 0.93 (0.70–1.24) |
| 432 | Occupations in IT-system-analysis, IT-application-consulting and IT-sales | 707 | 66.5% | 0.88 (0.84–0.93) | 0.89 (0.84–0.94) | 0.89 (0.84–0.93) | 0.89 (0.85–0.94) |
| 312 | Occupations in surveying and cartography | 161 | 67.7% | 0.90 (0.81–1.00) | 0.89 (0.80–0.99) | 0.89 (0.80–0.99) | 0.91 (0.82–1.01) |
| 514 | Service occupations in passenger traffic | 142 | 68.3% | 0.88 (0.79–0.98) | 0.89 (0.79–0.99) | 0.89 (0.79–0.99) | 0.85 (0.76–0.95) |
| 245 | Occupations in precision mechanics and tool making | 143 | 65.7% | 0.89 (0.79–1.00) | 0.89 (0.79–1.00) | 0.89 (0.79–1.00) | 0.89 (0.80–1.00) |
| 947 | Technical and management occupations in museums and exhibitions | 57 | 68.4% | 0.89 (0.74–1.06) | 0.89 (0.75–1.07) | 0.89 (0.75–1.07) | 0.90 (0.75–1.07) |
| 914 | Occupations in economics | 68 | 66.2% | 0.87 (0.73–1.03) | 0.88 (0.74–1.05) | 0.88 (0.74–1.04) | 0.88 (0.74–1.05) |
| 211 | Occupations in underground and surface mining and blasting engineering | 20 | 65.0% | 0.90 (0.65–1.24) | 0.88 (0.64–1.22) | 0.89 (0.64–1.22) | 0.89 (0.64–1.23) |
| 121 | Occupations in gardening | 325 | 66.2% | 0.88 (0.81–0.95) | 0.87 (0.81–0.95) | 0.88 (0.81–0.95) | 0.89 (0.82–0.96) |
| 421 | Occupations in geology, geography and meteorology | 116 | 65.5% | 0.86 (0.76–0.98) | 0.87 (0.76–0.99) | 0.87 (0.76–0.99) | 0.86 (0.76–0.98) |
| 117 | Occupations in forestry, hunting and landscape preservation | 86 | 64.0% | 0.86 (0.73–1.01) | 0.86 (0.73–1.00) | 0.86 (0.73–1.01) | 0.86 (0.74–1.01) |
| 931 | Occupations in product and industrial design | 43 | 65.1% | 0.85 (0.68–1.05) | 0.85 (0.69–1.05) | 0.85 (0.69–1.05) | 0.86 (0.69–1.06) |
| 511 | Technical occupations in railway, aircraft and ship operation | 45 | 62.2% | 0.84 (0.67–1.06) | 0.85 (0.68–1.06) | 0.85 (0.68–1.06) | 0.82 (0.65–1.02) |
| 243 | Occupations in treatment of metal surfaces | 19 | 63.2% | 0.86 (0.61–1.21) | 0.85 (0.61–1.18) | 0.85 (0.61–1.19) | 0.83 (0.59–1.16) |
| 411 | Occupations in mathematics and statistics | 109 | 62.4% | 0.82 (0.71–0.95) | 0.84 (0.73–0.97) | 0.84 (0.73–0.97) | 0.85 (0.74–0.98) |
| 422 | Occupations in environmental protection engineering | 48 | 64.6% | 0.85 (0.69–1.05) | 0.84 (0.68–1.03) | 0.84 (0.68–1.03) | 0.84 (0.69–1.03) |
| 434 | Occupations in software development and programming | 1,068 | 59.8% | 0.80 (0.76–0.84) | 0.80 (0.76–0.84) | 0.80 (0.76–0.84) | 0.81 (0.77–0.85) |
| 423 | Occupations in environmental protection management and environmental protection consulting | 93 | 60.2% | 0.79 (0.67–0.93) | 0.80 (0.67–0.94) | 0.80 (0.67–0.94) | 0.81 (0.68–0.95) |
| 524 | Ship’s officers and masters | 21 | 57.1% | 0.77 (0.53–1.11) | 0.77 (0.54–1.10) | 0.77 (0.54–1.11) | 0.72 (0.50–1.03) |
| 523 | Aircraft pilots | 30 | 50.0% | 0.68 (0.47–0.97) | 0.71 (0.50–1.01) | 0.71 (0.50–1.01) | 0.67 (0.47–0.96) |

Model estimates are presented as prevalence ratios (95% confidence intervals) and are arranged in descending order based on Model 2 results. Statistical results of occupations with fewer than ten cases of SP were not considered, including occupations in philology, occupations in industrial glass-making and -processing, occupations in musical instrument making, artisans designing ceramics and glassware, occupations in industrial ceramic-making and -processing, occupations in fishing, senior non-commissioned officers and higher, junior non-commissioned officers, occupations in vini- and viticulture. Missing values (n = 2,125) are not presented in the table.
