# Supplementary material for: Occupational differences in sickness presenteeism in the baseline survey of the German National Cohort (NAKO)
Source: J Occup Med Toxicol. 2026 Sep 25;21:31. doi: 10.1186/s12995-026-00529-1 (PMC13615606; doi:10.1186/s12995-026-00529-1)
Supplement: Supplementary file 4 [file 12995_2026_529_MOESM4_ESM.docx]

**Additional file 4:** Prevalence ratios (PRs) and 95% confidence intervals (CIs) for sickness presenteeism (SP) across occupational segments by supervisory/managerial status (full results)

| **Occupational segment** | **Supervisors and managers** | | **Non-supervisors and non-managers** | | **P** |
| --- | --- | --- | --- | --- | --- |
|  | **N (% SP)** | **Model 2 (main model)**  **PR (95% CI)** | **SP**  **N (%)** | **Model 2 (main model)**  **PR (95% CI)** |  |
|  |  | (adjusted for sex, age, educational level, health status) |  | (adjusted for sex, age, educational level, health status) |  |
| Safety and security occupations | 42 (90.5%) | 1.13 (1.03–1.25) | 2,229 (73.9%) | 1.00 (0.97–1.02) | 0.012 |
| Occupations in the food industry, in gastronomy and in tourism | 288 (85.4%) | 1.06 (1.00–1.11) | 1,730 (81.8%) | 1.06 (1.03–1.08) | 0.932 |
| Service occupations in social sector and cultural work | 684 (85.7%) | 1.05 (1.01–1.08) | 12,177 (80.0%) | 1.06 (1.05–1.07) | 0.513 |
| Occupations in commerce and trade | 844 (83.5%) | 1.04 (1.01–1.08) | 5,430 (80.3%) | 1.05 (1.04–1.07) | 0.641 |
| Medical and non-medical health care occupations | 339 (86.1%) | 1.04 (1.00–1.09) | 9,856 (82.5%) | 1.07 (1.06–1.09) | 0.186 |
| Occupations in building and interior construction | 473 (82.0%) | 1.03 (0.98–1.07) | 3,054 (72.5%) | 0.98 (0.96–1.00) | 0.044 |
| Occupations in traffic and logistics | 292 (81.8%) | 1.03 (0.98–1.09) | 3,798 (74.2%) | 0.99 (0.97–1.01) | 0.168 |
| Occupations concerned with production technology | 1,011 (79.7%) | 1.00 (0.96–1.03) | 7,466 (70.9%) | 0.95 (0.94–0.97) | 0.016 |
| Business related service occupations | 656 (78.7%) | 0.97 (0.93–1.01) | 12,334 (75.0%) | 0.97 (0.96–0.98) | 0.777 |
| Occupations in agriculture, forestry and horticulture | 58 (77.6%) | 0.97 (0.84–1.12) | 649 (70.4%) | 0.93 (0.88–0.98) | 0.556 |
| Occupations in business management and organisation | 1,449 (78.1%) | 0.96 (0.93–0.99) | 11,866 (75.5%) | 0.98 (0.97–0.99) | 0.178 |
| Manufacturing occupations | 171 (75.4%) | 0.95 (0.87–1.03) | 2,144 (71.8%) | 0.96 (0.93–0.98) | 0.809 |
| Service occupations in the IT-sector and the natural sciences | 324 (72.8%) | 0.91 (0.86–0.98) | 4,866 (67.3%) | 0.90 (0.88–0.92) | 0.582 |
| Occupations in cleaning services | 33 (69.7%) | 0.83 (0.67–1.04) | 467 (71.3%) | 0.92 (0.86–0.97) | 0.408 |

Model estimates are presented as prevalence ratios (95% confidence intervals) and are arranged in descending order based on Model 2 results for the manager subgroup.
